# Energetics of lipid transport by the ABC transporter MsbA is lipid dependent

**DOI:** 10.1038/s42003-021-02902-8

**Published:** 2021-12-09

**Authors:** Dawei Guo, Himansha Singh, Atsushi Shimoyama, Charlotte Guffick, Yakun Tang, Sam M. Rowe, Timothy Noel, David R. Spring, Koichi Fukase, Hendrik W. van Veen

**Affiliations:** 1 Department of Pharmacology, University of Cambridge, Tennis Court Road, Cambridge, CB2 1PD UK; 2 Department of Chemistry, Osaka University, 1-1 Machikaneyama, Toyonaka, Osaka 560-0043 Japan; 3 Department of Chemistry, University of Cambridge, Lensfield Road, Cambridge, CB2 1EW UK

**Keywords:** Membrane lipids, Enzyme mechanisms, Antimicrobial resistance

## Abstract

The ABC multidrug exporter MsbA mediates the translocation of lipopolysaccharides and phospholipids across the plasma membrane in Gram-negative bacteria. Although MsbA is structurally well characterised, the energetic requirements of lipid transport remain unknown. Here, we report that, similar to the transport of small-molecule antibiotics and cytotoxic agents, the flopping of physiologically relevant long-acyl-chain 1,2-dioleoyl (C18)-phosphatidylethanolamine in proteoliposomes requires the simultaneous input of ATP binding and hydrolysis and the chemical proton gradient as sources of metabolic energy. In contrast, the flopping of the large hexa-acylated (C12-C14) Lipid-A anchor of lipopolysaccharides is only ATP dependent. This study demonstrates that the energetics of lipid transport by MsbA is lipid dependent. As our mutational analyses indicate lipid and drug transport via the central binding chamber in MsbA, the lipid availability in the membrane can affect the drug transport activity and vice versa.

## Abstract (web summary)

Guo et al. conduct a biochemical analysis of the energy dependence of substrate transport by MsbA, an essential ABC lipid transporter in Gram-negative bacteria. The authors examine the flopping of physiologically relevant lipids by MsbA and their differing dependencies on ATP and chemical proton gradient, demonstrating that the energetics of lipid transport by MsbA is lipid dependent.

## Introduction

The passive movement of phospholipids between the leaflets in a membrane bilayer is extremely slow, in the order of hours to days, as shown in model membranes^1,2^. This is, at least in part, due to the energetically unfavourable movement of the hydrophilic headgroup across the hydrophobic core of the bilayer. For this reason, the cell requires membrane-embedded enzymes that facilitate the transbilayer movement of lipids within seconds. Examples of mammalian phospholipid transporters include (i) the calcium-activated nhTMEM16 scramblase that catalyses the shuffling of phospholipids between the inner and outer leaflets of the plasma membrane independent of metabolic energy^3^, (ii) the type IV P-type ATPase flippases that catalyse the inward translocation of phospholipids from the outer leaflet to the inner leaflet of the plasma membrane^4^, and (iii) the liver ATP-binding cassette (ABC) floppase ABCB4^5^ for which in vivo data indicate a role in the outward translocation of phosphatidylcholine (PC) and its biliary secretion from hepatocytes into the canaliculi^6^. Phospholipid transport is equally important in microorganisms. The dimeric ABC exporter MsbA is an essential lipid transporter in Gram-negative bacteria. In vivo observations point to a role of MsbA in the translocation of phospholipids and core Lipid-A from their point of synthesis at the inner leaflet of the plasma membrane to the outer leaflet^7,8^. In further steps at the outer leaflet of the plasma membrane, a polysaccharide moiety (O-antigen) is ligated to core Lipid-A to form full-length lipopolysaccharides (LPS). The Lipid-A (endotoxin) domain of LPS is a unique, glucosamine-based phospholipid that serves as the hydrophobic anchor of LPS and is the bioactive component of the molecule that is associated with Gram-negative septic shock. After it is formed, LPS is transported across the periplasm into the outer leaflet of the outer membrane by the LPS translocase LptB2FGCADE^9^. But exactly how MsbA mediates the transport of naturally abundant lipids and how this reaction is related to MsbA’s ability to transport amphiphilic drugs^10–15^ has not been investigated in detail. Here we study the energetics of lipid transport by purified MsbA in proteoliposomes and compare the lipid and drug transport activities in a mutagenesis approach.

## Results

### Energetics of lipid floppase activity

**Fig. 1:**
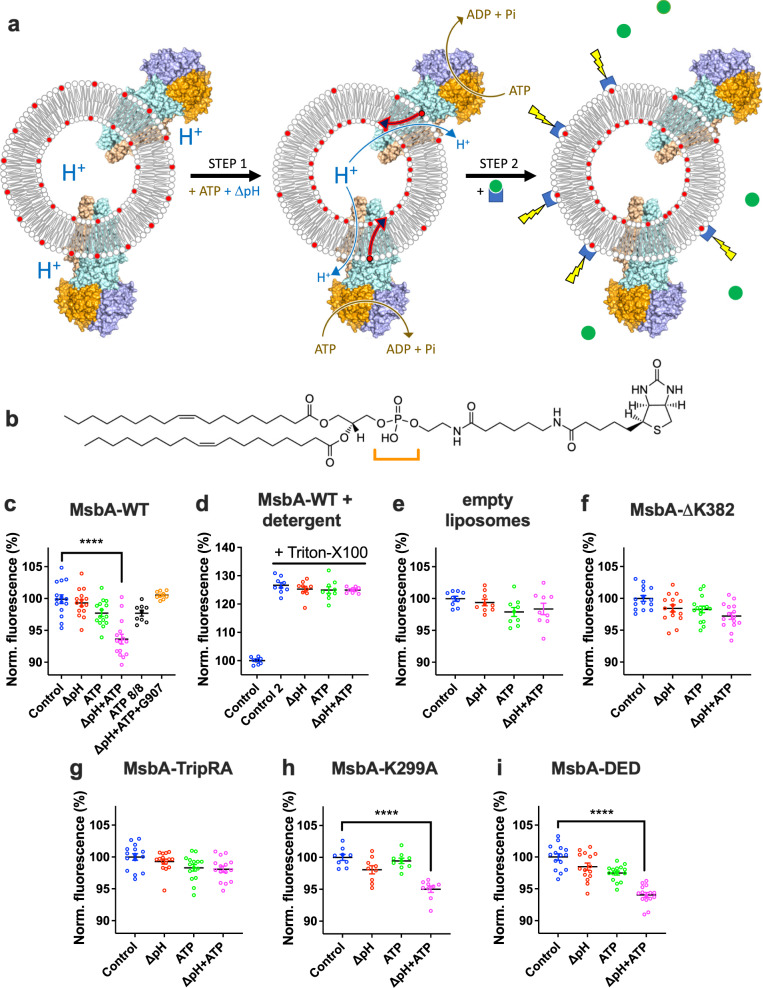
PE transport by MsbA. **a** Schematic showing proteoliposomes containing headgroup-labelled biotin-PE (headgroup depicted as red circle with two C18 acyl chains in grey) in the inner and outer leaflet of the membrane, and the MsbA homodimer inserted in an inside-out fashion. Step 1, the PE transport reaction (red arrow) by MsbA is initiated by the addition of Mg-ATP in the external buffer and the imposition of a ΔpH (interior acidic) across the membrane. ATP binding and hydrolysis (brown arrow) at the nucleotide-binding domain dimer (in orange and slate blue) and simultaneous proton conduction (blue arrow) by the membrane domains (MD) (in light orange and light blue) provide metabolic energy for the transport of PE from the outer leaflet of the membrane to the inner leaflet. Step 2, following transport activity, the remaining amount of biotin-PE in the outer leaflet is quantified from the fluorescence emission (yellow lightning bolts) of fluorescence-tagged avidin (blue concave rectangles) when a bound quencher (green circles) is displaced by the binding of the biotin moiety. **b** Structural formula of the 1,2-dioleoyl (C18) biotin-PE in which the orange bracket highlights the phosphodiester moiety. **c**, **d** Biotin-PE transport assays with MsbA-WT before (**c**) or after (**d**) the addition of 1% (v/v) detergent Triton X-100 at the end of the transport reaction. Note the change in the fluorescence scale in **d**. Data refer to different provisions of metabolic energy: (i) Control (no metabolic energy, pH_in_ 6.8/pH_out_ 6.8) (set at 100%), (ii) imposed ΔpH (pH_in_ 6.8/pH_out_ 8.0), (iii) ATP (pH_in_ 6.8/pH_out_ 6.8), (iv) imposed ΔpH (pH_in_ 6.8/pH_out_ 8.0) plus ATP, or (v) ATP 8/8 (pH_in_ 8.0/pH_out_ 8.0). Control 2 in **d** refers to the Control after Triton X-100 addition. The MsbA inhibitor G907 was used in **c**. **e** Biotin-PE transport assays with liposomes without MsbA proteins. **f**–**i** Biotin-PE transport assays with ATPase-deficient MsbA-ΔK382 (**f**) and substrate binding chamber mutants MsbA-TripRA (R78A R148A R296A) (**g**), MsbA-K299A (**h**) and MsbA-DED (D41N E149Q D252N) (**i**). Data represent observations in three experiments (*n* = 3) with independently prepared batches of proteoliposomes. Lines and error bars in scatter dot plots refer to mean ± s.e.m. (one-way analysis of variance; ****P < 0.0001). Asterisks above the square brackets refer to comparisons with the control without metabolic energy.

**Fig. 2:**
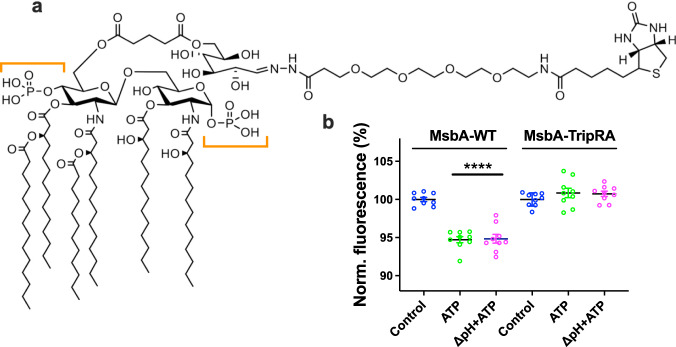
Lipid-A transport by MsbA. **a** Structural formula of biotin-Lipid-A containing four primary hydroxymyristate (C14) acyl chains and one secondary myristate and laurate (C12) acyl chain. The orange brackets highlight the two phosphate groups in the glucosamine moieties of the lipid. **b** Biotin-Lipid-A assays with MsbA-WT and substrate binding chamber mutant MsbA-TripRA (R78A R148A R296A). Data refer to different provisions of metabolic energy: (i) Control (no metabolic energy, pH_in_ 6.8/pH_out_ 6.8), (ii) ATP (pH_in_ 6.8/pH_out_ 6.8) or (iii) imposed ΔpH (pH_in_ 6.8/pH_out_ 8.0) plus ATP. Data represent observations in three experiments (*n* = 3) with independently prepared batches of proteoliposomes. Lines and error bars in the scatter dot plot refer to mean ± s.e.m. (one-way analysis of variance; ****P < 0.0001). Asterisks above the horizontal line refer to comparisons with the control without metabolic energy.

We conveniently express *Escherichia coli* MsbA in *Lactococcus lactis*, a Gram-positive bacterium that lacks Lipid-A and LPS as well as an *E. coli*-like periplasm and outer membrane^10,11,15^. We use these cells as a source of MsbA for lipid transport studies in proteoliposomes (Fig. 1). As (i) a previous study detected the transport of phospholipids and Lipid-A by MsbA in a ^32^Pi and ^14^C-acetate labelling approach in *E. coli* cells^16^, (ii) PE is the predominant phospholipid in *E. coli* making up 70–80% of the total phospholipid pool^17,18^, and (iii) advanced studies by native mass spectrometry detected the binding of PE to MsbA^19^, we established a transport assay for physiologically relevant long-acyl-chain (2 × C18) PE in proteoliposomes in which MsbA was incorporated in an inside-out fashion (Fig. 1a). Lipids labelled with fluorescent nitrobenzoxadiazole (NBD) have frequently been used in transport studies with ABC transporters^20,21^ and P-type ATPases^22^. However, as MsbA mediates the efflux of fluorescent dyes^10,11,13,15^, this ABC transporter might interact with NBD-lipid as a drug analogue rather than a lipid. Moreover, NBD-labelled lipids are usually detected using fluorescence quenchers such as Co^2+^ and dithionite, the intrinsic membrane permeability of which reduces the ability to discriminate between labelled lipids in the outer and inner leaflet of the membrane. In our assay, 1 in 17 PE molecules in the proteoliposomes contained a non-invasive biotin moiety that was covalently linked to the ethanolamine head group of the lipid (Fig. 1b). We detected PE flopping by inside-out oriented MsbA as a relocation of the lipid from the outer to the inner leaflet of the membrane. The reduction in the amount of external biotin-PE over time was quantified from the emission of membrane-impermeable fluorescence-tagged avidin when a quencher in complex with the avidin is displaced by the biotin moiety of the PE (Fig. 1a). The potential sources of metabolic energy that can drive substrate transport by MsbA, ATP and the transmembrane chemical H^+^ gradient (∆pH), were applied using well-established methods^15^. Briefly, proteoliposomes prepared in buffer pH 6.8 were diluted in buffer pH 8.0, imposing a difference between the interior pH and external pH by a pH jump (pH_in_ 6.8/pH_out_ 8.0) that was stabilised by the dissociation of NH_4_^+^ in the lumen of proteoliposomes and the outward diffusion of NH_3_. Using the fluorescent pH indicator BCECF trapped in the proteoliposomal lumen, measurements of the interior pH over time demonstrate that this pH difference was sustained for the 20 min duration of the biotin-lipid floppase assays (Supplementary Fig. 1). Where required, ATP was included in the external buffer. Remarkably, a significant PE floppase activity for MsbA-WT was observed only in the simultaneous presence of 5 mM Mg-ATP and the ∆pH (pH_in_ 6.8/pH_out_ 8.0) (Fig. 1c). The increased biotin-PE signal obtained with the addition of Triton-X100 to disrupt these proteoliposomes at the end of the floppase assay (Fig. 1d), demonstrates that PE flopping is based on the translocation of lipid to the inner leaflet of the membrane. This conclusion is strengthened by the finding that free biotin is neither transported by MsbA, nor liberated from biotin-PE by spontaneous hydrolysis during the floppase assay (Supplementary Fig. 2). No significant PE floppase activity was observed in MsbA-WT-containing proteoliposomes in the presence of ATP without the ΔpH (pH_in_ 6.8/pH_out_ 6.8 and pH_in_ 8.0/pH_out_ 8.0) or the ΔpH (pH_in_ 6.8/pH_out_ 8.0) only (Fig. 1c), indicating the importance of the simultaneous presence of both forms of metabolic energy. Furthermore, PE floppase activity was not detected in empty liposomes (Fig. 1e) or proteoliposomes containing MsbA-ΔK382 that lacks the catalytic Walker A lysine residue and has strongly reduced ATPase activity^11^ (Fig. 1f). A previous cryo-electron microscopic (cryo-EM) study identified the quinoline compound G907 as a specific MsbA inhibitor that binds to and stabilises the inward-facing MsbA dimer, thereby preventing the transition to the outward-facing conformation^23^. We synthesised G907 (Supplementary Methods) and tested its efficacy in our biotin-PE transport assay. At a 100 nM concentration (5x the reported in-vitro IC_50_^23^), G907 completely inhibited active biotin-PE transport in MsbA-WT-containing proteoliposomes (Fig. 1c), thus confirming that this activity is mediated by MsbA. These findings indicate that inside-out oriented MsbA transports biotin-PE from the outer leaflet to the inner leaflet of the proteoliposomal membrane. This floppase activity is based on apparent H^+^/biotin-PE antiport and is facilitated by ATP binding and hydrolysis in the presence of a ΔpH (interior acidic in the proteoliposomes). The lipid transport assays were repeated using the relatively large *E. coli* hexa-acylated (C12-C14) Lipid-A that is biotin-labelled on the disaccharide core (Fig. 2a)^24^. Different from biotin-PE, significant biotin-Lipid-A transport was observed in the proteoliposomes with ATP only; this activity was not affected by the imposed ΔpH (interior acidic) (Fig. 2b and Supplementary Fig. 1). Therefore, the transport of Lipid-A and PE has different energetic requirements.

### Similar pathways for lipids and drugs

**Fig. 3:**
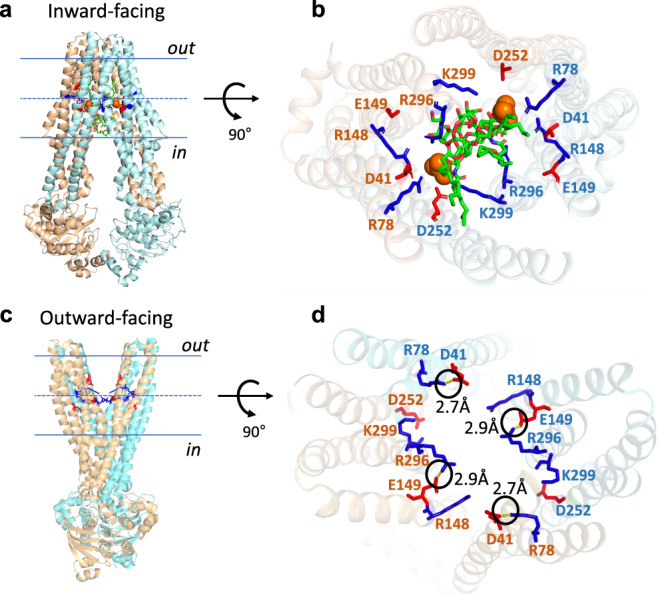
Structure models of inward-facing MsbA with bound Lipid-A and outward-facing MsbA without the lipid. **a** Ribbon diagram of inward-facing MsbA with bound Lipid-A (PDB-ID: 5TV4) shows the location of arginine residues R78, R148, R296, K299 and carboxyl residues D41, E149 and D252 (referred to as the ‘ring’ residues) in the substrate binding chamber near the centre of phospholipid bilayer. View in the plane of the plasma membrane with the two half-transporters in light blue and light orange. **b** Close-up view from the external face of the membrane shows how the arginine and carboxyl side chains (in blue and red stick representation) in the MD of each half-transporter form a ring of hydrophilic interactions with the 1 and 4′ phosphate group (in orange spheres) and the glucosamine, ester and amide groups connecting the acyl chains in Lipid-A (in green stick representation). The colours of the residue numbers refer to the half-transporters in which the residues are located. The biotin tag in the disaccharide core of our biotin-Lipid-A (Fig. 2a) does not interfere with the interactions of Lipid-A with the ring residues. **c** Ribbon diagram of outward-facing MsbA (PDB-ID: 3B60) from which Lipid-A has dissociated, shown with the same arginine and carboxyl residues as in **a**. **d** Close-up view from the external face of the membrane shows that R78-D41 and R296-E149 form salt-bridges within each half-transporter (indicated by circles with distance in Å). Figure was generated in PyMol v2.4.1.

**Fig. 4:**
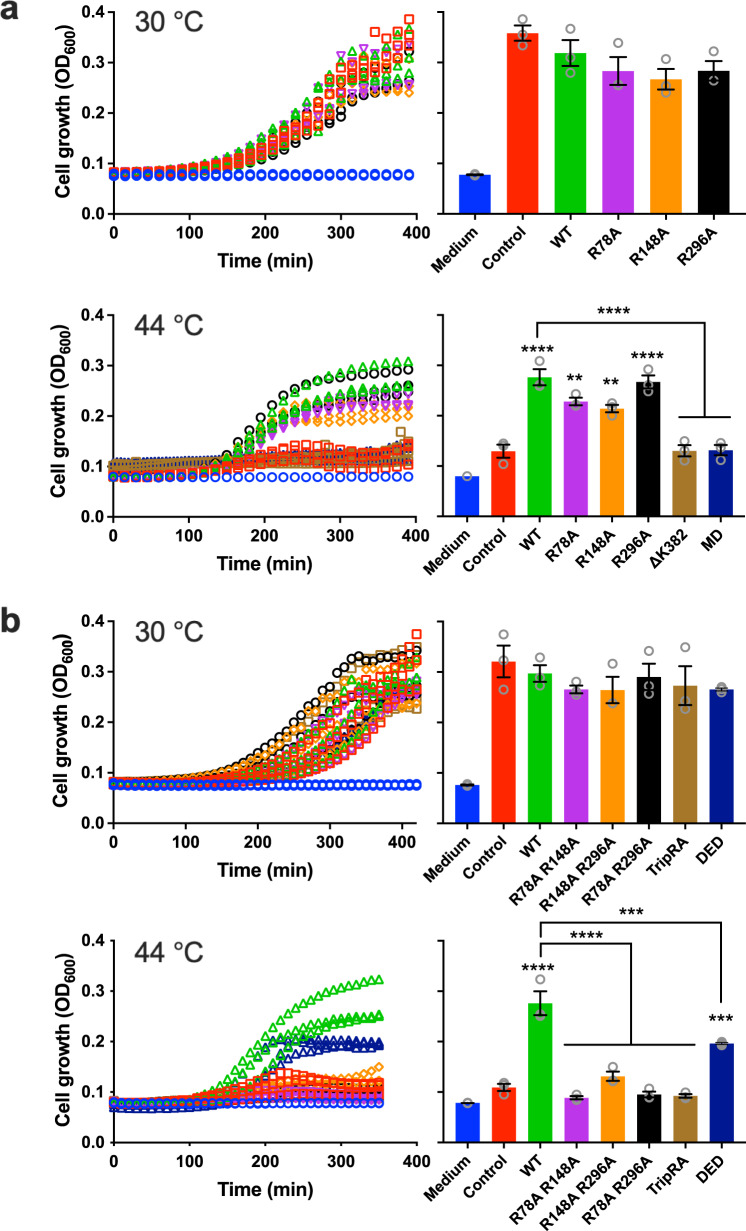
Lipid-A and phospholipid transport by MsbA proteins in *E. coli* cells. *E. coli* WD2 expresses a genome-encoded temperature-sensitive MsbA protein containing an alanine to threonine substitution at position 270 in TMH5^16^. At non-permissive temperature (44 °C), this MsbA mutant rapidly inactivates. Due to a deficiency in the MsbA-mediated transport of phospholipids and Lipid-A to the outer membrane, the biogenesis of the outer membrane is impaired, prohibiting cell growth and division^7,41^. In contrast, *E. coli* WD2 cells exhibit normal growth at 30 °C. To test whether the expression of plasmid-encoded MsbA proteins can rescue the *E. coli* WD2 cells at 44 °C, the cells were transformed with pBAD24-based plasmids containing wild-type or mutant *msbA* genes. Medium without cells (Medium) (blue) and cells with empty plasmid (Control) (red) served as controls. **a** Expression of MsbA-WT (green), single substrate binding chamber mutants R78A (magenta), R148A (orange) or R296A (black), ATPase-deficient MsbA-ΔK382^11^ (light brown) or drug transport-active MsbA-MD lacking the nucleotide-binding domain^15^ (dark blue). **b** Expression of MsbA-WT (green), double mutants R78A R148A (magenta), R148A R296A (orange) or R78A R296A (black), MsbA-TripRA (R78A R148A R296A) (light brown) or triple substrate-binding chamber mutant MsbA-DED (D41N E149Q D252N) (dark blue). Histograms show OD_600_ levels near 375 min; bar colours match those in the traces. The results demonstrate that ATP hydrolysis at the nucleotide-binding domains and arginine and carboxyl side chains in the central binding cavity are important for in vivo lipid transport by MsbA. All mutants were equally well expressed as MsbA-WT in the plasma membrane (Supplementary Figs. 3 and 4). Data points represent observations in three experiments (*n* = 3) with independently prepared batches of cells. Values in histograms show mean ± s.e.m. (one-way analysis of variance; **P < 0.01; ***P < 0.001; ****P < 0.0001). In the data for 44 °C, the asterisks above bars refer to comparison with the non-expressing control, whereas the asterisks above the square brackets refer to comparisons with MsbA-WT.

Recent cryo-EM and crystal structures of the MsbA-Lipid-A complex suggest that R78 (transmembrane helix (TMH) 2), R148 (TMH3), Q256 (TMH5), R296, K299 (TMH6) and interspersed carboxyl residues D41 (TMH1), E149 (TMH3) and D252 (TMH5) form a ring of polar interactions with the 1 and 4′ phosphate group and the glucosamine, ester and amide groups connecting the acyl chains in Lipid-A^8,23,25^ (Fig. 3). Similar acidic and basic residues coordinate Lipid-A binding in the cryo-EM structure of the LPS translocase LptB2FGC^26^. We tested the effect of mutations of the ‘ring’ residues in MsbA on lipid and drug transport. First, we focussed on the arginine residues in the ring and found a deficiency in active Lipid-A and PE transport in proteoliposomes for a triple mutant MsbA containing R78A R148A R296A substitutions (referred to as MsbA-TripRA) (Figs. 1g and 2b); the K299A mutation did not inhibit PE transport (Fig. 1h). Moreover, we tested the impact of the arginine substitutions on MsbA-mediated lipid transport in *E. coli* WD2 cells. These cells contain a genome-encoded MsbA mutant A270T that misfolds at non-permissive temperature (44 °C), causing a strong deficiency in cell growth. This phenotype could be rescued by the expression of plasmid-encoded MsbA-WT or single arginine substitution mutants but not by the (i) double arginine mutants R78A R148A, R78A R296A, R148A R296A, (ii) triple arginine mutant MsbA-TripRA, (iii) ATPase-deficient MsbA-ΔK382 or (iv) truncated MsbA-MD protein lacking the nucleotide-binding domain (Fig. 4). All mutants were expressed at a comparable level as MsbA-WT in the plasma membrane of *E. coli* WD2 cells (Supplementary Figs. 3 and 4). These in vivo experiments point to a requirement for two out of the three arginine side chains and functional nucleotide-binding domains in lipid transport by MsbA.

**Fig. 5:**
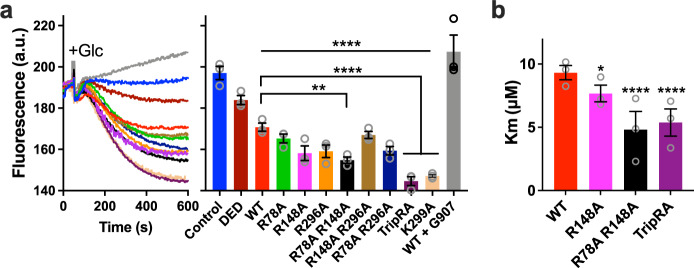
Ethidium transport by MsbA in *L. lactis*. **a** ATP-depleted cells were preloaded with 2 µM ethidium after which ethidium efflux was initiated by the addition of glucose (+Glc) as a source of metabolic energy^11,15^. Active efflux by MsbA-WT was abolished in the presence of 2.5 µM of the MsbA inhibitor G907 (0.7x the reported in-vivo IC_50_ in cells^23^). MsbA-mediated ethidium efflux was inhibited by the triple DED mutations in the substrate binding chamber and was enhanced by K299A and sequential R-to-A replacements in the chamber. Fluorescence traces are the mean of three independent experiments. Values in histogram show significance of fluorescence levels near 600 s. All mutants were equally well expressed as MsbA-WT in the plasma membrane (Supplementary Figs. 5 and 6). **b** Effect of R-to-A mutations on the apparent affinity (*K*_m_) of MsbA for ethidium in the transport reaction. Histogram data represent observations in three experiments (*n* = 3) with independently prepared batches of cells and are expressed as mean ± s.e.m. (one-way analysis of variance; *P <0.05; **P < 0.01; ****P < 0.0001). Asterisks above the square brackets refer to comparisons with the MsbA-WT, whereas asterisks above the horizontal line refer to comparisons with the non-expressing control.

In addition to lipids, MsbA transports small molecules like ethidium, erythromycin and chloramphenicol^10,11,15^. The efflux of ethidium can conveniently be monitored in *L. lactis* cells from the decrease in fluorescence emission when the dye dissociates from intracellular nucleic acids^27^. In contrast to the observations for Lipid-A and PE, the R78A R148A double mutant and TripRA mutant showed a significantly enhanced efflux of ethidium compared to MsbA-WT (Fig. 5a). Analysis of protein expression levels and the rate of ethidium transport as a function of the dye concentration identified an increased ethidium binding affinity of MsbA as the underlying mechanism for the enhanced efflux activity (Fig. 5b and Supplementary Figs. 5 and 6). Hence, the arginine side chains facilitate the binding of Lipid-A and PE in the chamber but inhibit the binding of ethidium.

Second, we focussed on carboxyl residues D41, E149 and D252 that, in inward-facing MsbA, are interspersed between R78, R148 and R296 and located in the vicinity of the polar headgroup of bound Lipid-A (Fig. 3). In our lipid transport assays, we observed that the D41N, E149Q and D252N triple mutation (referred to as MsbA-DED) is tolerated in biotin-PE transport in proteoliposomes (Fig. 1i). However, MsbA-DED showed considerably reduced lipid transport in *E. coli* WD2 cells at non-permissive temperature (Fig. 4b) suggesting that the carboxylates are important for Lipid-A transport. MsbA-DED is also impaired in ethidium efflux in cells (Fig. 5a), which agrees with a previous study where the DED mutation was found to strongly inhibit ATP-dependent ethidium^+^/H^+^ antiport by MsbA^15^. When taken together, these data indicate that ethidium, biotin-PE and biotin-Lipid-A are transported via the central substrate binding chamber in which the basic residues R78, R148 and R296 and acidic residues D41, E149 and D252 promote interactions of MsbA with Lipid-A. The basic residues are important for the interactions with PE but inhibit the interactions with cationic ethidium. Instead, ethidium transport is facilitated by the presence of the acidic residues.

## Discussion

During cell growth and division of Gram-negative bacteria, phospholipids and core Lipid-A are synthesised in the inner leaflet of the plasma membrane and exported (flopped) by MsbA to the outer leaflet, from where they are transported by the LPS translocase LptB2FGCADE^9,26^ to the expanding outer membrane. Structural studies suggest that MsbA is a Lipid-A floppase but no direct biochemical measurements of the flopping of Lipid-A with purified MsbA have been reported to date. Consequently, the energetic requirements of the MsbA-mediated lipid transport reactions have not been studied in detail. These measurements are technically challenging because there is no net change in the amount of lipid in the membrane; the lipid reorients from one side to the other.

In our Lipid-A and PE transport assays, we measured the metabolic energy-dependent floppase activity of purified MsbA in proteoliposomes, prepared with *E. coli* lipids and egg yolk PC, as a relocation of biotinylated lipid from the external membrane surface of proteoliposomes to the internal membrane surface. The biotin-lipid remaining in the outer leaflet of the membrane is detected by the binding of membrane-impermeable avidin (Fig. 1a). Control experiments show that free biotin is neither transported by MsbA, nor liberated from the biotinylated lipids in the time course of the assays (Supplementary Fig. 2). In further control experiments, the lipid floppase activity (i) does not occur in empty liposomes but requires functional MsbA protein, (ii) is inhibited by the MsbA inhibitor G907, and (iii) is affected by the ring residues in the substrate binding chamber that are known to coordinate Lipid-A binding (Figs. 1–5). The location of the biotin tag in the disaccharide core of our biotin-Lipid-A (Fig. 2a) ensures that the biotin does not interfere with the interactions of Lipid-A with the ring residues (Fig. 3a, b). Given the biotin-labelling of 1 in 17 PE molecules in our proteoliposomal membranes, the excess of unlabelled PE over biotin-Lipid-A, and the chemical differences between the biotinylated lipids and the native lipids they mimic, the measured PE and Lipid-A transport activities most likely underestimate the in-situ activity of MsbA as a lipid floppase.

**Fig. 6:**
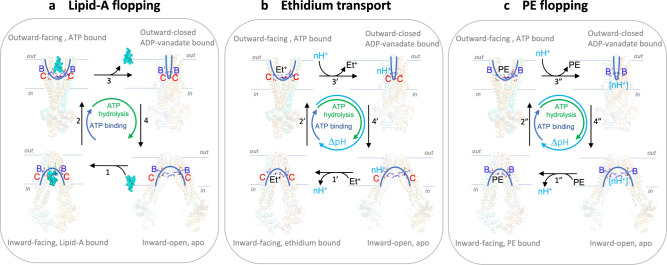
Schematic on the energetics of lipid and drug transport by MsbA. **a** In Lipid-A flopping, the arginine side chains of R78, R148 and R296 (indicated by blue B) and interspersed acidic side chains of D41, E149 and D252 (indicated by red C) in the central binding chamber (indicated by half blue ovals) coordinate a multitude of polar interactions with the two glucosamine and phosphate moieties of Lipid-A (in cyan blue). The sequence of reaction steps 1–4, underlying the alternating access of the binding chamber, is dependent on ATP binding and hydrolysis in our experiments and is based on structures for inward-open apo MsbA (PDB-ID: 3B5W), inward-facing G907-Lipid-A-bound MsbA (PDB-ID: 6BPL) and outward-closed ADP-vanadate-bound MsbA (PDB-ID: 5TTP) from *E. coli* and outward-facing AMP-PNP-bound MsbA from *Salmonella typhimurium* (PDB-ID: 3B60). Out and in refer to the outside and inside of the plasma membrane. **b** Ethidium transport is ATP binding and hydrolysis and ΔpH (interior alkaline)-dependent. Deprotonation of the C residues in inward-facing conformation in Step 1′ is followed by ethidium (Et^+^) binding near these residues. The B residues are not essential in this reaction and are omitted in the schematic. Step 2′, Transition to outward-facing state. Step 3′, Ethidium release and protonation of C residues. Step 4′, ΔpH-dependent transition to inward-facing state. **c** In PE flopping, Steps 1”–4” represent analogous ATP and ΔpH-dependent reactions as shown for ethidium, in which the B residues coordinate the binding of the phosphodiester group in PE. As the C residues are not essential in our PE transport measurements, proton coupling will involve protonatable groups (indicated by [nH^+^]) other than D41, E149 and D252.

The findings in this study represent important advancements in our understanding of lipid transport by MsbA. First, the process of Lipid-A transport from the plasma membrane to the outer membrane in *E. coli* requires two major transport systems, MsbA and LptB2FGCADE, the activity of which could be intrinsically connected. As summarised in the schematic in Fig. 6a–c, our results demonstrate that MsbA can mediate the transport of Lipid-A and PE in the absence of auxiliary proteins. Therefore, MsbA functions as an independent lipid floppase in the plasma membrane that supports LptB2FGCADE, and possibly also the MlaFEDB phospholipid translocase^28–30^, in anterograde lipid trafficking from the outer leaflet of the plasma membrane to the outer membrane. The amount of LPS, and its underacylated forms^31^, versus phospholipid that is ultimately transported to the outer membrane might, therefore, be regulated at the level of MsbA. Second, we show that the activity of ATP-dependent biotin-PE transport in proteoliposomes is strongly stimulated by the imposition of a ΔpH (interior acidic) (Figs. 1 and 6c). The repetition of these measurements with long-acyl-chain (2 × C18) NBD-PE also demonstrates the importance of the ΔpH + ATP in the MsbA-mediated transport of this lipid (Supplementary Fig. 7), and extends previous observations on the low ATP-dependent transport activity of MsbA for long-acyl-chain NBD-PE in proteoliposomes^32^. As the efficient transport of ethidium, chloramphenicol and erythromycin by MsbA requires the input of ATP and ΔpH (Fig. 6b), in which the ATPase reaction is stimulated by the ΔpH^15^, the energetics of PE transport is most similar to that of small-molecule transport. On the other hand, the Lipid-A transport reaction is ATP-dependent without a noticeable contribution of the ΔpH (Figs. 2 and 6a). The requirement for ATP binding and hydrolysis in Lipid-A and PE transport is in agreement with the phospholipid and Lipid-A-stimulated ATPase activity of purified MsbA^33^ and the dimerisation of the nucleotide-binding domains in the MsbA dimer upon Lipid-A binding^13^. We conclude that the role of the ΔpH in ATP-dependent lipid transport by MsbA is independent of the biotin modification but is linked to the structural properties of the transported lipid. Third, our data suggest that lipids and drugs share similar pathways in MsbA. Both types of substrates are transported via the central substrate binding chamber of MsbA where they interact with the same ring of side chains in the substrate binding chamber. R78, R148, R296, K299, and interspersed D41, E149 and D252 are known to form hydrophilic interactions with the two anionic phospho-glucosamine moieties in Lipid-A in inward-facing MsbA and to rotate away in the outward-facing state, thus enabling electrostatic interactions between R78-D41 and R296-E149 and facilitating lipid release (Fig. 3). These basic and acidic residues are important for Lipid-A transport in our experiments (Figs. 2, 4 and 6a). The arginine residues in the ring also support the transport of the smaller PE ligand that contains a single phosphodiester moiety but no glucosamine groups (Figs. 1 and 6c). In agreement with the PE data, basic residues contribute to the coordination of the phosphodiester moiety of phospholipid substrates in the crystallised lipoxygenase enzyme LOX^34^ and periplasmic phospholipid transport protein Ttg2D^35^ from *Pseudomonas aeruginosa*. Reported molecular dynamics simulations for the phospholipid scramblase nhTMEM16 from *Nectria haematococca* also point to a role of basic residues in the coordination of phospholipid binding^36^. Although the R78, R148, R296 residues in the ring are essential for PE transport, they are inhibitory for ethidium transport. In particular, the ethidium efflux rate and apparent binding affinity increased with the removal of basic residues in the MsbA-R78A R148A and MsbA-TripRA mutants (Fig. 5). The replacement of K299 in the ring by A (MsbA-K299A) showed a similar enhancement of ethidium transport (Fig. 5a) but did not affect the transport of PE (Fig. 1h). The binding sites for PE and ethidium are, therefore, partially overlapping. As our biotin-PE contains C18 acyl chains, whereas structural evidence suggests that the interior binding chamber can accommodate Lipid-A with C12 and C14 acyl chains^23^, the possibility exists that, similar to small molecules, only the headgroup of biotin-PE is accommodated in the central binding chamber. The acyl chains would remain in the phospholipid bilayer. Related mechanisms were proposed for phospholipid flipping by eukaryotic type IV P-type ATPases^4,37^ and the transport of lipid-linked oligosaccharide by the ABC floppase PglK from *Campylobacter jejuni*^38^. However, with two acyl chains in PE versus 6 acyl chains in Lipid-A, the acyl chain region in PE is much less densely packed and, due to the rotational freedom around the *sp3* hybridised carbon atoms, it might have sufficient conformational flexibility to be accommodated in the chamber.

In the transport of ethidium by MsbA, the carboxyl residues in the ring are essential for transport activity (Fig. 5). We suggest that, following the binding of cationic ethidium near the ring carboxylates in the inward-facing state, proton binding to these carboxylates in the outward-facing conformation aids in the dissociation of the bound ethidium towards the extracellular environment and the reorientation of MsbA to the inward-facing state (Fig. 6b). The transport cycle is completed by the deprotonation of the carboxyl residues in the cellular interior and binding of ethidium. In this way, the ATP-dependent ethidium transport reaction in cells is facilitated by the imposition of the ΔpH (interior alkaline) across the plasma membrane. Proton coupling in ATP-dependent biotin-PE transport will involve protonatable groups other than D41, E149 and D252.

In conclusion, our findings demonstrate MsbA-mediated Lipid-A and PE transport with different energy requirements and provide important details in the structural mechanisms proposed for MsbA based on X-ray crystallography and cryo-EM techniques. MsbA transports lipids and amphiphilic drugs out of cells by shared translocation pathways. The lipid availability in the membrane can, therefore, affect the drug transport activity and vice versa. This conclusion will also be relevant for other multidrug transporters that can transport drugs and lipids as alternative substrates.

## Materials and methods

### Compound synthesis

The synthesis of biotin-labelled *E. coli* Lipid-A from the glutaryl glucose linker-containing Lipid-A derivative and biotin-dPEG4^TM^-hydrazide (Invitrogen) is described by Fujimoto et al.^24^. The selective MsbA inhibitor G907 was synthesised by the procedure reported by Ho et al.^23^ and is described in full in the Supplementary Methods.

### Bacterial strains, mutagenesis and protein expression

The drug-hypersensitive *L. lactis* NZ9000 Δ*lmrA* Δ*lmrCD*^39^, lacking expression of the endogenous multidrug transporters LmrA and LmrCD, was used for the expression of His_6_-tagged MsbA proteins from nisin-inducible pNZ8048 plasmids in the nisin controlled expression (NICE) system (NIZO Food Research, The Netherlands)^40^. The expression of MsbA proteins from arabinose-inducible pBAD24 plasmid (ATCC product 87399) in *E. coli* WD2 cells is described below under ‘Cell growth experiments’.

For site-directed mutagenesis of MsbA, the pGEM-5Zf(+) (Promega)-derived cloning vector pGEM-MsbA containing coding regions for an amino-terminal His_6_-tag and thrombin cleavage site followed by the wild-type *E. coli msbA* gene with silenced internal NcoI sites (at position 595 and 1375)^10,11^, was propagated in *E. coli* XL1-Blue cells (New England Biolabs) grown in Luria-Bertani Broth medium supplemented with 100 µg/mL ampicillin. Mutant *msbA* genes were generated in pGEM-MsbA by round-the-horn mutagenesis using the following primers: R78A, forward primer 5′-GCCGGTATCACCAGCTATGTCTCCAGC-3′, reverse primer 5′-TAAAATCATCAGCCCGATCACC-3′; R148A, forward primer 5′-GCAGAAGGTGCGTCGATCATCG-3′, reverse primer 5′-CACAACAGTAATCAGTGCGCC-3′; R296A, forward primer 5′-GCCCCGCTGAAATCGCTGACC-3′, reverse primer 5′-CATCAGTGCAATCATTGAAGAGAAAAC-3′; K299A, forward primer 5′-GCTTCGCTGACCAACGTTAACGCC-3′, reverse primer 5′-CAGCGGACGCATCAGTGCAATCA-3′. The mutant *msbA* genes were sequenced to ensure that only the intended changes had been introduced. Similar to wild-type *msbA* and previously described mutants^10,11,15^, the mutant genes were subcloned as NcoI/XbaI fragments into linearised pNZ8048 and pBAD24 plasmid DNA.

Overnight cultures of the lactococcal cells containing pNZ plasmid encoding (i) wild-type MsbA (referred to as MsbA-WT)^10^, (ii) MsbA-MD lacking the nucleotide-binding domain^15^, (iii) the single MsbA mutants: ΔK382^11^, K299A, R78A, R148A, or R296A, (iv) the double mutants: R78A R148A, R78A R296A, or R148A R296A, or (v) the triple mutants: R78A R148A R296A (referred to as MsbA-TripRA) or D41N E149Q D252N (referred to as MsbA-DED)^15^ were diluted 1:25 (v/v) in M17 medium (Formedium) supplemented with 25 mM glucose and 5 µg/mL chloramphenicol. Cells were allowed to grow at 30 °C to an OD_660_ of 0.55–0.60, after which 10 pg mL^−1^ nisin-A^11^ was added to induce protein expression for 1 h.

### Preparation of inside-out membrane vesicles

Lactococcal cells from 2 L culture were harvested at 4 °C by centrifugation (13,000 × *g*, 10 min). The cell pellet was washed once (100 mM KP_i_, pH 7.0) and resuspended in 25 mL of the same buffer. Chicken egg white lysozyme (3 mg mL^−1^, Sigma-Aldrich) was added together with half a tablet of Complete-Protease Inhibitor Cocktail (Sigma-Aldrich) and the mixture was incubated for 30 min at 30 °C. To lyse the cells, the mixture was passaged twice through a cell disrupter (Basic Z 0.75-kW Benchtop Cell Disrupter, Constant Systems) at 20,000 p.s.i. Subsequently, 10 μg mL^−1^ DNase and 10 mM MgSO_4_ were added, and the resultant mixture was incubated for 30 min at 30 °C to digest DNA and RNA. In all, 15 mM K-EDTA (pH 7.0) was then added, and the mixture was centrifuged for 40 min at 13,000 × *g* and 4 °C. The supernatant containing the membrane vesicles was transferred to a clean tube and centrifuged for 1 h at 125,000 × *g* and 4 °C. The membrane vesicle pellet was resuspended in 50 mM KP_i_ buffer (pH 7.0) containing 10% (v/v) glycerol to a total membrane protein concentration of 60 mg mL^−1^. The membrane vesicle suspension was stored in aliquots in liquid nitrogen. The presence of MsbA proteins in membrane vesicles was assessed on western blot probed with a 1:1000 (v/v) dilution of primary mouse anti-polyhistidine tag antibody (Sigma-Aldrich, cat. no.: H1029) followed by a 1:5000 (v/v) dilution of horseradish peroxidase-conjugated secondary goat anti-mouse antibody (Sigma-Aldrich, cat. no.: A4416). The histidine-tagged MsbA signals were detected by chemiluminescence using SuperSignal™ West Pico PLUS Chemiluminescent Substrate (ThermoFisher Scientific) and Odyssey Fc Imaging System (Li-Cor Biosciences).

### Purification of His-tagged MsbA proteins

Histidine-tagged MsbA proteins were purified by Ni^2+^-nitrilotriacetic acid (NTA) affinity chromatography. In a typical experiment, 200 µg nickel-Ni^2+^-NTA resin with a binding capacity of more than 15 mg mL^−1^ and bead size between 45 and 165 µm, was pre-equilibrated by washing 5 times with 5 resin volumes of ultrapure water and twice with 5 resin volumes of wash Buffer A (50 mM KP_i_ (pH 8.0), 0.1 M NaCl, 10% (v/v) glycerol, 0.05% (w/v) n-dodecyl-β-D-maltoside (DDM) and 20 mM imidazole (pH 8.0)). After every wash, the resin was collected by centrifugation (175 × *g*, 1 min, 4 °C). To solubilise the target protein, 600 µL membrane vesicles (36 mg total protein) were thawed and added to 4 mL of solubilisation buffer (50 mM KP_i_ (pH 8.0), 10% (v/v) glycerol, 0.1 M NaCl and 1% (w/v) DDM). The mixture was shaken gently at 4 °C for 4 h. The mixture was added to 200 μg washed Ni^2+^-NTA resin and the suspension was mildly rotated overnight at 4 °C. The resultant resin with bound protein was transferred to a 1.5 mL bed-volume disposable Biospin chromatography column (Bio-Rad), and was washed with 20 resin volumes of wash Buffer A and 30 resin volumes of Wash Buffer B (50 mM KP_i_ (pH 7.0), 0.1 M NaCl, 10% (v/v) glycerol, 0.05% (w/v) DDM and 20 mM imidazole (pH 8.0)). Subsequently, the His-tagged protein was eluted with 500 μL of elution buffer (50 mM KP_i_ (pH 7.0), 0.1 M NaCl, 5% (v/v) glycerol, 0.05% (w/v) DDM and 150 mM imidazole (pH 8.0)). The purified protein was kept on ice and used for further experiments immediately.

### Preparation of proteoliposomes containing biotinylated lipids

Purified MsbA proteins were reconstituted in proteoliposomes in an inside-out fashion^15^. For this purpose, 8 mg of liposomes were prepared from (i) acetone-ether-washed total lipid extract from *E. coli*, (ii) egg yolk phosphatidylcholine, and (iii) 18:1 Biotinyl Cap PE (1,2-dioleoyl-sn-glycero-3-phosphoethanolamine-*N*-(cap biotinyl), referred to as biotin-PE) (all purchased from Avanti Polar Lipids Inc.) mixed at a ratio of 3:1:0.12 (w/w/w) in chloroform. For the preparation of biotin-Lipid-A containing proteoliposomes, the biotin-PE was replaced by 0.5% (w/w) biotin-Lipid-A^24^ (stored at 0.5 mg mL^−1^ in chloroform:methanol 9:1 (v/v)). The solvent was evaporated from the lipid mixture by flushing with N_2_ gas after which the lipid mixture was hydrated in Buffer 1 (20 mM KP_i_, 100 mM NH_4_SCN, 50 mM K_2_SO_4_, pH 6.8) at a concentration of 4 mg mL^−1^. Using a LiposoFast-Basic extruder (Avestin), lipids were extruded 11 times through a 400 nm polycarbonate filter to form unilamellar liposomes of homogenous size. The extruded liposomes were destabilised by adding Triton X-100 until the maximum OD_540_ was just passed. For reconstitution, 160 µg of purified protein was mixed with destabilised liposomes in a ratio of 1:50 (w/w) to a final concentration of 80 µg mL^−1^. The mixture was left shaking gently at RT for 30 min. To remove detergent, the proteoliposomes were successively incubated with polystyrene bio-beads (Bio-Bead SM-2, Bio-Rad): 80 mg mL^−1^ at room temperature for 2 h, 8 mg mL^−1^ at 4 °C for 2 h, and finally 8 mg mL^−1^ at 4 °C for 18 h. Before use, the bio-beads were pre-washed 3 times with methanol, once with ethanol, four times with ultrapure water and once with Buffer 1. Ready-made proteoliposomes were harvested by centrifugation at 165,000 × *g* and 4 °C for 20 min and resuspended in 200–300 μL Buffer 1. Samples were kept on ice and immediately used in further experiments.

### Biotin–lipid floppase assays

MsbA-containing proteoliposomes and empty liposomes containing 3% (w/w) biotin-PE or 0.5% (w/w) biotin-Lipid-A were diluted 6-fold in Buffer 1 in which they were prepared (no gradient control) or Buffer 2 (20 mM KP_i_, 100 mM KSCN, pH 8.0) to impose a ΔpH (interior acidic). All buffers were pre-warmed at 30 °C and supplemented with 5 mM MgSO_4_ and, where indicated, 5 mM Mg-ATP. Following dilution, the proteoliposomes were incubated for 20 min at 30 °C. Subsequently, 9 to 15 samples of 10 μL were each mixed with 90 µL working solution of Pierce™ Fluorescence Biotin Quantitation Kit. The mixture was incubated at RT for 10 min, and the fluorescence intensity was measured in a CLARIOstar plate reader (BMG Labtech) with excitation and emission wavelength of 494 nm and 520 nm, respectively. For biotin-Lipid-A analyses, the *F*_p(e)_/mean *F*_p(c)_ ratio was divided by the *F*_l(e)_/mean *F*_l(c)_ for each data point × 100%, where p corresponds to MsbA-containing proteoliposomes, l corresponds to empty liposomes (without MsbA protein), e corresponds to the treatment with ATP or ΔpH+ATP and c corresponds to the control treatment without metabolic energy. For biotin-PE analyses, *F*_l(e)_ = *F*_l(c)_ in the ΔpH, ATP and ΔpH+ATP treatments. Therefore, we directly compared *F*_p(e)_/mean *F*_p(c)_ ratios for the different treatments, and the mean *F*_p(c)_ was set at 100%.

### Internal pH measurements in proteoliposomes

Proteoliposomes were prepared by the same protocol as described under ‘Preparation of proteoliposomes containing biotinylated lipids’. However, 100 μM 2′,7′-bis-(2-carboxyethyl)−5-(and-6)-carboxyfluorescein (BCECF) was added to Buffer 1 in which the lipids were hydrated before extrusion. The BCECF-loaded proteoliposomes were 100-fold diluted in 2 mL of prewarmed Buffer 1 or Buffer 2, with a composition as described under ‘Biotin–lipid floppase assays’, in a 3.5 mL glass cuvette. BCECF fluorescence was monitored in a LS 55B Luminescence Spectrometer (PerkinElmer) at excitation and emission wavelengths of 502 and 525 nm, and with slit widths of 5 nm each.

### Cell growth experiments

*E. coli* WD2 cells expressing a chromosome-encoded temperature-sensitive MsbA A270T mutant^7,41^ were used to study Lipid-A transport by pBAD24 plasmid-encoded MsbA proteins. The cells were grown aerobically overnight at 30 °C in LB broth supplemented with 12.5 µg mL^−1^ tetracycline and 100 µg mL^−1^ ampicillin. The cell cultures were first diluted in antibiotic-containing LB medium and grown to an OD_600_ of 0.6–0.7. The cell cultures were then diluted 500-fold in fresh antibiotic-containing LB broth containing 0.02% (w/v) of the inducer arabinose, of which 300 µL aliquots were dispensed in the wells on a 96-well plate. To assess cell growth, the OD_600_ of the cell suspensions was monitored at 44 or 30 °C for 6.5 h in a CLARIOstar plate reader in absorbance mode at 600 nm.

### Ethidium transport

To measure ethidium efflux, 50 mL cultures of *L. lactis* cells with or without expressed MsbA proteins were harvested by centrifugation at 4 °C (6500 × *g*, 10 min) and washed once with wash buffer (50 mM KP_i_, 5 mM MgSO_4_, pH 7.0). Cells were de-energised in the presence of 0.5 mM 2,4-dinitrophenol at 30 °C for 40 min, and washed 3 times with the wash buffer, and resuspended to an OD_660_ of 5. For each measurement, 200 μL of cell suspension was diluted in 1800 μL of pre-warmed wash buffer at 30 °C in a 3.5 mL glass cuvette. Ethidium bromide (2 μM) was added to the mixture and the fluorescence level was monitored in a LS 55B Luminescence Spectrometer (PerkinElmer) at excitation and emission wavelengths of 500 and 580 nm with slit widths of 10 and 5 nm, respectively. Fluorescence traces were collected in BioLight Studio FL v1.04.02. Ethidium efflux was initiated by the addition of 25 mM glucose after which the ethidium fluorescence intensity was monitored for a further 10 min. The apparent affinity of MsbA proteins for ethidium in the transport reaction was determined as described^10,11^, by measuring the ethidium concentration dependence of the rate of MsbA-facilitated ethidium uptake into de-energised cells. The rates in MsbA-expressing cells were corrected for passive ethidium influx in de-energised control cells without MsbA expression. For the determination of kinetic parameters (*K*_m_ and *V*_max_) the data were fitted to the Michaelis–Menten equation in Graphpad Prism v 9.2.0.

### NBD-PE floppase assay

MsbA-containing proteoliposomes and empty liposomes were prepared as described under ‘Preparation of proteoliposomes containing biotinylated lipids’ using a lipid mixture composed of (i) acetone-ether-washed total lipid extract from *E. coli*, (ii) 18:1 phosphatidylglycerol (1,2-dioleoyl-sn-glycero-3-phospho-(1′-rac-glycerol)) and (iii) 18:1 headgroup-labelled NBD-PE (1,2-dioleoyl-sn-glycero-3-phosphoethanolamine-*N*-(7-nitro-2-1,3-benzoxadiazol-4-yl) at a ratio of 3: 1: 0.012 (w/w/w). In the floppase assay, the (proteo)liposomes were diluted 100-fold in 2 mL prewarmed Buffer 1 or Buffer 2, with a composition as described under ‘Biotin–lipid floppase assays’, in a 3.5 mL glass cuvette. Following dilution, the proteoliposomes were incubated for 5 min at 30 °C. Subsequently, 4 mM sodium dithionite was added to quench the NBD fluorescence in the outer leaflet of the membrane. The remaining NBD fluorescence in the inner leaflet was measured in a LS 55B Luminescence Spectrometer (PerkinElmer) at excitation and emission wavelengths of 464 and 536 nm with slit widths of 10 and 5 nm, respectively. Fluorescence traces were collected in BioLight Studio FL v1.04.02. For analysis, the fluorescence intensity was normalised to *F*/*F*_max_ and, following the addition of the dithionite, the (*F*/*F*_max_)_p_/(*F*/*F*_max_)_l_ ratios at plateau for the different treatments (ATP, ΔpH, and ΔpH+ATP) were compared with the control without metabolic energy (set at 100%), where p corresponds to MsbA-containing proteoliposomes, l corresponds to liposomes without MsbA protein, and *F*_max_ and *F* correspond to the observed fluorescence plateau before and after dithionite addition.

### Statistics and reproducibility

All experiments were performed at least three times using three independent batches of cells and proteoliposomes. Significance of data was tested by one-way analysis of variance and Tukey’s multiple group comparisons. Asterisks shown in histograms and scatter dot plots either refer to comparisons with control (above the bar) or comparisons of specific pairs (horizontal brackets): *P < 0.05; **P < 0.01; ***P < 0.001; ****P < 0.0001.

### Reporting summary

Further information on research design is available in the Nature Research Reporting Summary linked to this article.

## Supplementary information

Supplementary Information

Description of Additional Supplementary Files

Supplementary Data 1

Reporting Summary

## Data Availability

Data that support the findings of this study have been deposited in the University of Cambridge research repository Apollo with link 10.17863/CAM.75215 (ref. ^42^) or are available from the corresponding author upon reasonable request. The source data for the figures and supplementary figures are included in Supplementary Data 1. The DNA sequences of pNZ-MsbA, pGEM-MsbA and pBAD-MsbA plasmid and the wild-type and mutant *msbA* genes, as well as the amino acid sequences of the MsbA proteins, are included in Supplementary Data 1. Requests for unique materials should be addressed to the corresponding author. The pNZ plasmids utilise the nisin controlled expression (NICE) system, which was obtained from NIZO Food Research, The Netherlands.
