# Supplementary material for: Energetics of lipid transport by the ABC transporter MsbA is lipid dependent
Source: Commun Biol. 2021 Dec 9;4:1379. doi: 10.1038/s42003-021-02902-8 (PMC8660845; doi:10.1038/s42003-021-02902-8)
Supplement: Supplementary file 1 — Supplementary Information [file 42003_2021_2902_MOESM1_ESM.pdf]

SUPPLEMENTARY INFORMATION

Energetics of lipid transport by the ABC transporter MsbA is lipid dependent

Dawei Guo,[1] Himansha Singh,[1] Atsushi Shimoyama,[2] Charlotte Guffick,[1] Yakun Tang,[1] Sam M. Rowe,[3] Timothy Noel,[1] David R. Spring,[3] Koichi Fukase,[2] and Hendrik W. van Veen[1]*

[1]Department of Pharmacology, University of Cambridge, Tennis Court Road, Cambridge CB2 1PD, UK.

[2]Department of Chemistry, Osaka University, 1-1 Machikaneyama, Toyonaka, Osaka, 560-0043, Japan.

[3]Department of Chemistry, University of Cambridge, Lensfield Road, Cambridge CB2 1EW, UK.

*Corresponding author. **Email:** hwv20@cam.ac.uk (H.W.V.V.)

SUPPLEMENTARY FIGURES 1 – 7, page 2 - 9

SUPPLEMENTARY METHODS, page 10 - 27

SUPPLEMENTARY REFERENCES, page 28

SUPPLEMENTARY FIGURES

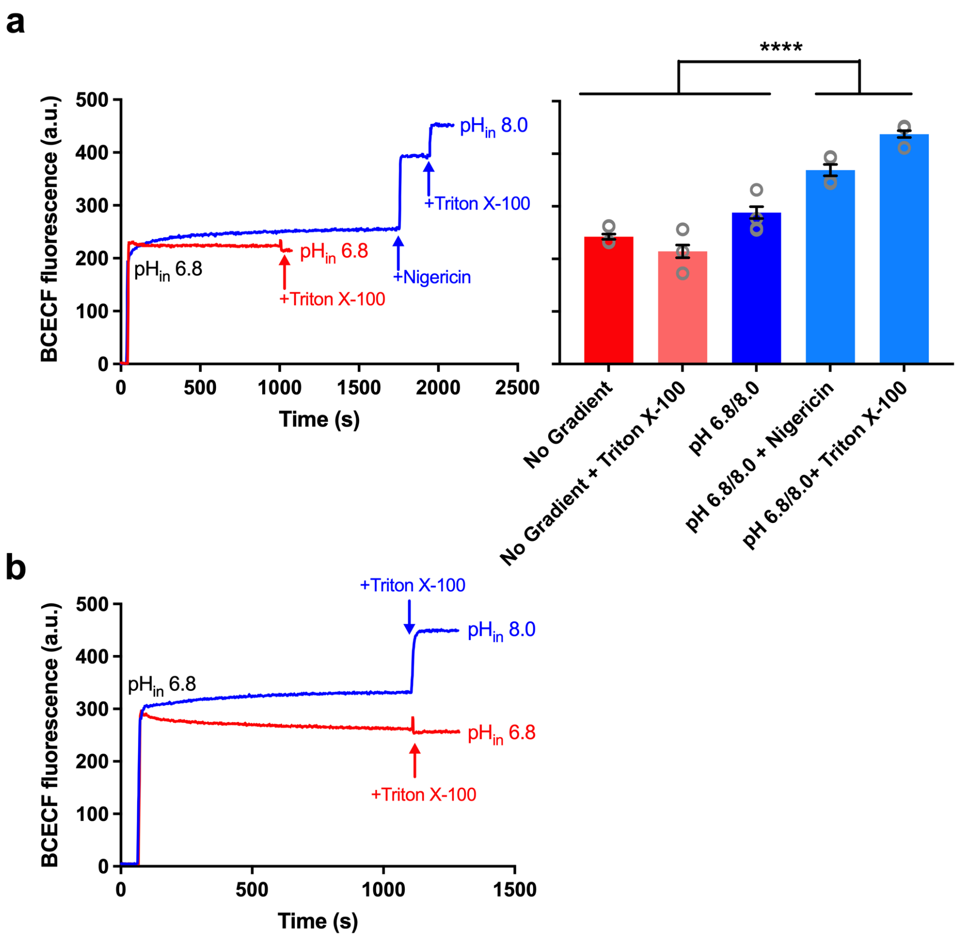

Supplementary Figure 1. Stability of the imposed ΔpH in MsbA-containing proteoliposomes. a, b Proteoliposomes containing MsbA-WT and 3% biotin-PE (**a**) or MsbA-WT and 1% biotin-Lipid-A (**b**), and with a pH_{in} of 6.8, were loaded with the fluorescent pH indicator BCECF (apparent pKa 6.98) and diluted 100-fold in buffer with pH 8.0 to impose a ΔpH (pH_{in} 6.8/pH_{out} 8.0) (blue trace) or in buffer with pH 6.8 as a control (pH_{in} 6.8/pH_{out} 6.8) (red trace). At the arrows, 2 μM of the ionophore nigericin or 0.025 % (v/v) Triton X-100 was added to increase the proton permeability of the proteoliposomal membrane, allowing protons to diffuse from the acidic interior to the alkaline exterior. Values in the histogram in (**a**) show fluorescence levels as mean ± s.e.m (n = 3). Asterisks above the square bracket show significant differences following the increase in the proton permeability of the proteoliposomes, indicating the stability of the imposed pH gradient during the biotin-PE and biotin-Lipid-A floppase assays (one-way analysis of variance; ****P＜0.0001).

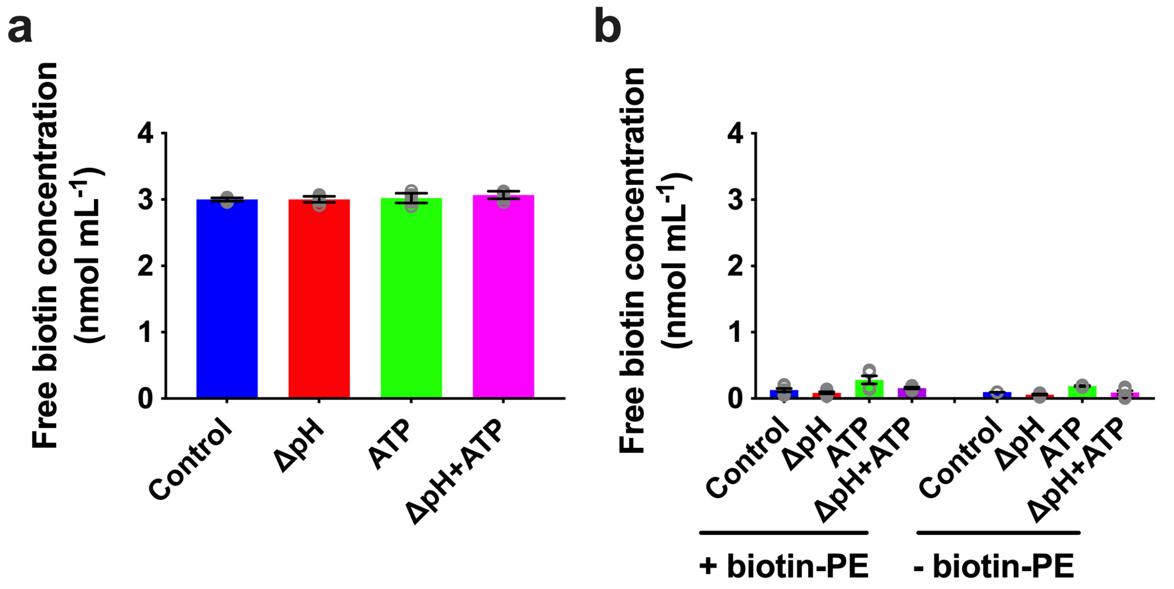

Supplementary Figure 2. MsbA does not transport free biotin in the biotin-lipid transport assays. a Proteoliposomes without biotin-PE received 3 μM free biotin before the start of the transport reaction, which yields a very similar fluorescence level as the standard amount of biotin-PE in the proteoliposomes. Reactions were started with the provision of different combinations of metabolic energy: (i) control (pH_{in} 6.8/pH_{out} 6.8), (ii) imposed ΔpH (pH_{in} 6.8/pH_{out} 8.0), (iii) ATP (pH_{in} 6.8/pH_{out} 6.8), and (iv) ATP plus imposed ΔpH (pH_{in} 6.8/pH_{out} 8.0). After the reaction time was completed, the free biotin concentration in the external buffer was measured. No significant differences in biotin concentrations were observed, suggesting that MsbA does not mediate the uptake of free biotin in the proteoliposomes. **b** Identical transport assays as in (**a**) were performed with biotin-PE-containing proteoliposomes. The proteoliposomes were then spun down by centrifugation, after which the supernatant was used to measure the concentration of free biotin liberated from the biotin-PE by the end of the transport assay. No significant differences were found for the various incubations, or when compared with proteoliposomes lacking biotin-PE. This result indicates that the biotin-PE is not hydrolysed into free biotin and PE over the time course of the transport experiments. Values in the histograms represent observations in three experiments (n = 3) with independently prepared batches of proteoliposomes and are expressed as mean ± s.e.m (one-way analysis of variance; not significant).

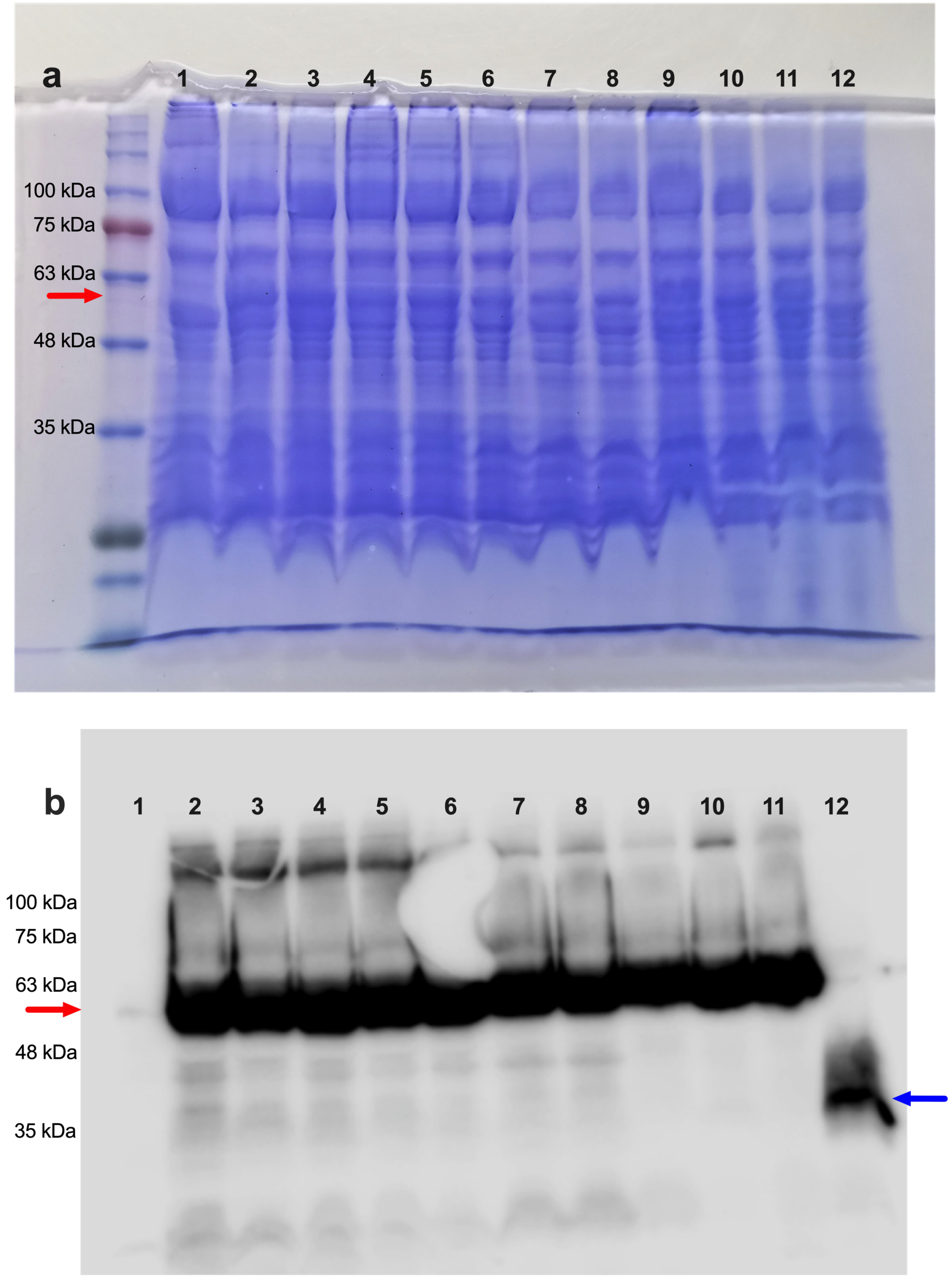

Supplementary Figure 3. Expression of MsbA proteins in *E. coli* WD2. a Coomassie brilliant blue-stained SDS-PAGE gel showing total membrane proteins without MsbA expression (*lane 1*) or with expression of MsbA-WT (*lane 2*), single MsbA mutants R78A (*lane 3*), R148A (*lane 4*), R296A (*lane 5*), double MsbA mutants R78A R148A (*lane 6*), R148A R296A (*lane 7*), R78A R296A (*lane 8*), MsbA-TripRA (*lane 9*), MsbA-DED (*lane 10*), MsbA-ΔK382 (*lane 11*) or MsbA-MD (*lane 12*). On the left side of lane 1, the positions of molecular mass markers are shown. **b** Western blot probed with anti-His tag antibody. MsbA proteins are indicated by the red arrows. MsbA-MD is indicated by the blue arrow.

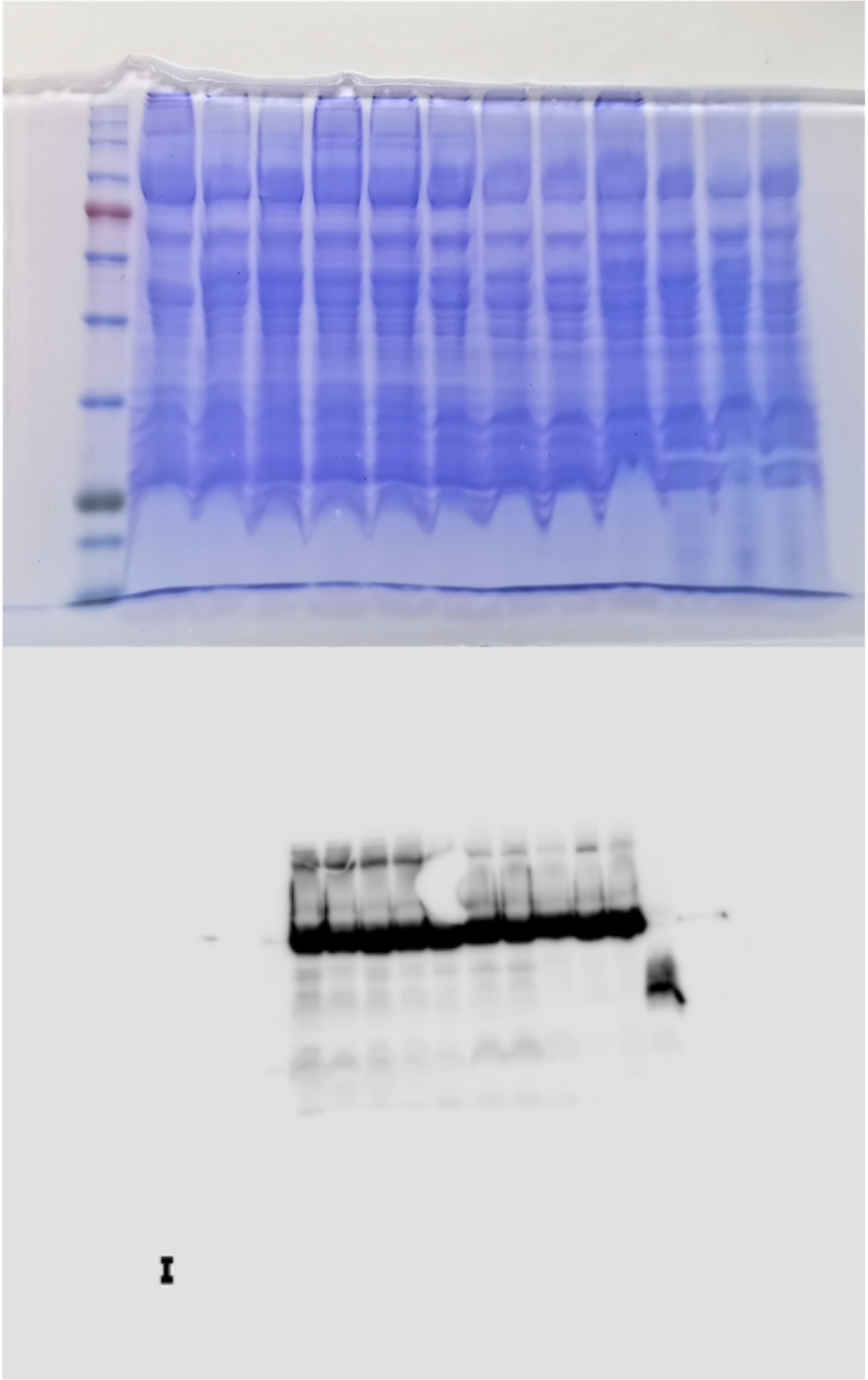

Supplementary Figure 4. Unprocessed SDS-PAGE and immunoblot used in Supplementary Fig. 3.

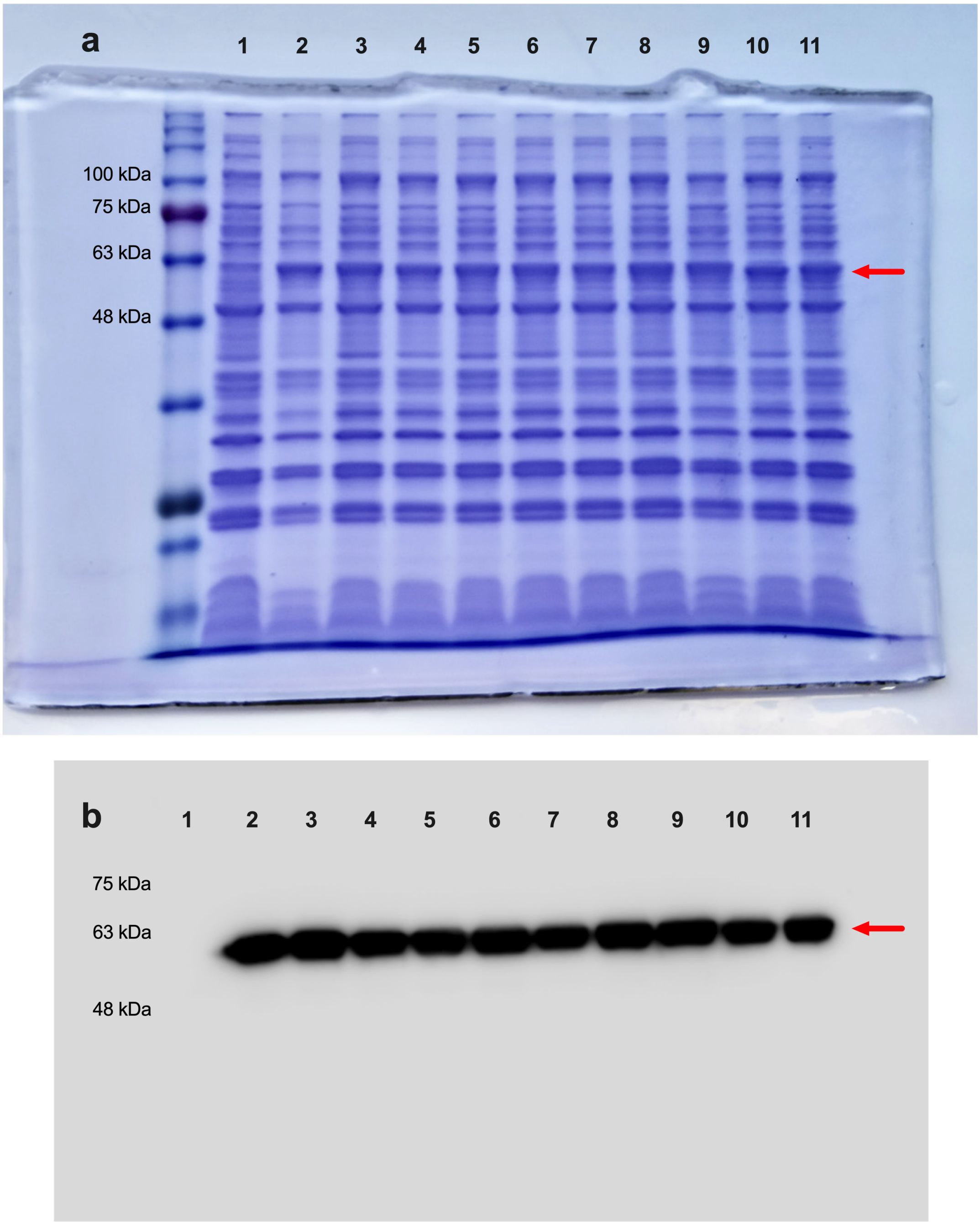

Supplementary Figure 5. Expression of MsbA proteins in *L. lactis*. a Coomassie brilliant blue-stained SDS-PAGE gel showing total membrane proteins of *L. lactis* without MsbA expression (*lane 1*) or with expression of MsbA-WT (*lane 2*), single MsbA mutants R78A (*lane 3*), R148A (*lane 4*), R296A (*lane 5*), double MsbA mutants R78A R148A (*lane 6*), R148A R296A (*lane 7*), R78A R296A (*lane 8*), MsbA-TripRA (*lane 9*), MsbA-DED (*lane 10*) or MsbA-ΔK382 (*lane 11*). Densitometric analysis (ImageJ 1.52t) shows that all mutants were expressed at a level of approx. 20% of total membrane protein, as previously reported for MsbA-WT [1]. On the left side of lane 1, the positions of molecular mass markers are shown. **b** Western blot probed with anti-His tag antibody. MsbA proteins are indicated by red arrows.

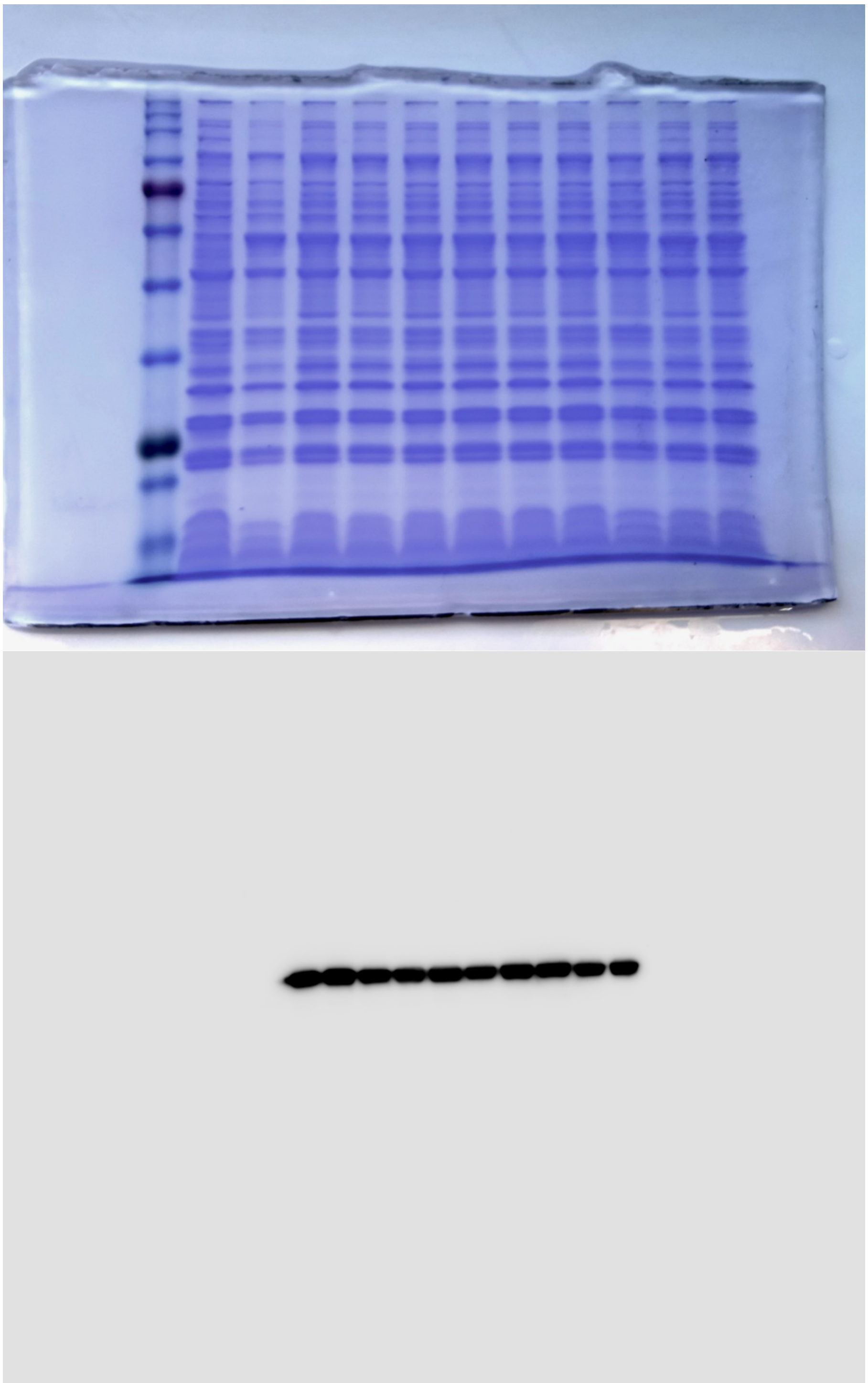

Supplementary Figure 6. Unprocessed SDS-PAGE and immunoblot used in Supplementary Fig. 5.

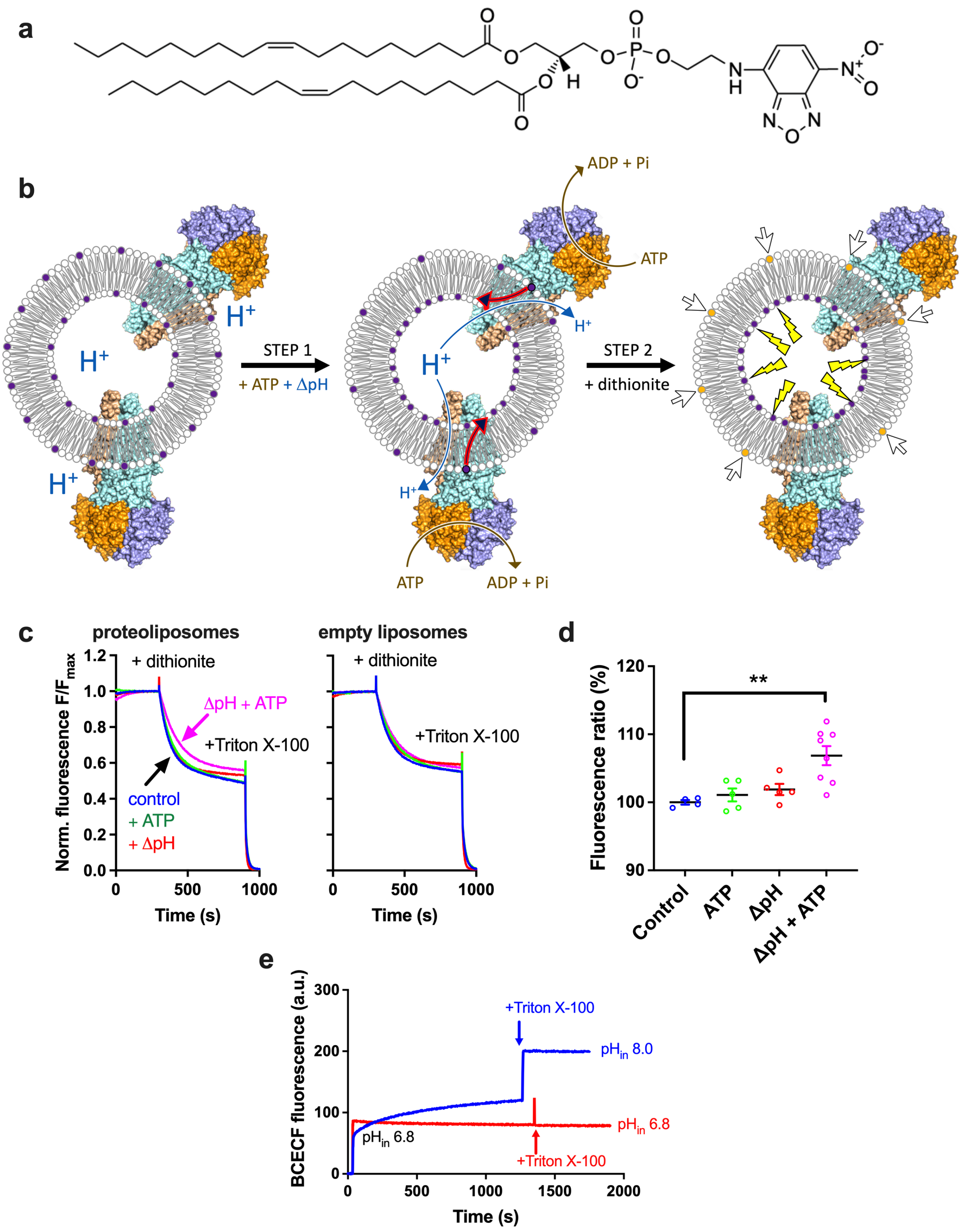

Supplementary Figure 7. NBD-PE transport by MsbA. a Structural formula of 1,2-dioleoyl (C18) NBD-PE. **b** Schematic showing proteoliposomes containing headgroup-labelled NBD-PE (headgroup depicted as purple circle with two C18 acyl chains in grey) in the inner and outer leaflet of the membrane, and the MsbA homodimer inserted in an inside-out fashion as in Fig. 1. *Step 1*, the NBD-PE transport reaction (red arrow) by MsbA is initiated by the addition

of Mg-ATP in the external buffer and the imposition of a ΔpH (interior acidic) across the membrane. *Step 2*, following lipid transport for 5 min, the fluorescence emission of NBD-PE in the outer leaflet of the membrane is quenched (indicated by the orange circles and white arrows) via chemical reduction of the fluorophore by dithionite (4 mM) in the external buffer. **c** Representative traces in the floppase assay with MsbA-containing proteoliposomes and empty liposomes in the presence of different sources of metabolic energy. The addition of dithionite (to quench NBD fluorescence in the outer leaflet of the membrane) and Triton X-100 (to quench all NBD fluorescence in permeabilised membrane) is indicated. F corresponds to the fluorescence intensity measured for each time point. F_{max} is the average fluorescence measured during the first 250s. **d** Following the addition of dithionite, the NBD-PE fluorescence in the inner leaflet of the membrane (yellow lightning bolts in (**b**)) at the plateau is plotted as the fluorescence ratio of F/F_{max} for proteoliposomes over F/F_{max} for empty liposomes. The results demonstrate that NBD-PE flopping by MsbA is stimulated by the simultaneous presence of ATP and the imposed ΔpH (pH_{in} 6.8/pH_{out} 8.0) compared to the control without metabolic energy (pH_{in} 6.8/pH_{out} 6.8) (set at 100%) or incubations with imposed ΔpH (pH_{in} 6.8/pH_{out} 8.0) or ATP only. Lines and error bars in the scatter dot plot refer to mean ± s.e.m. and represent observations with independently prepared batches of proteoliposomes (n = 4, or more). Asterisks above the square bracket refer to comparison with the control without metabolic energy (one-way analysis of variance; $^{**}P < 0.01$). **e** pH_{in} measurements in proteoliposomes (pH_{in} 6.8/pH_{out} 8.0 in blue and pH_{in} 6.8/pH_{out} 6.8 in red) with the fluorescent pH indicator BCECF demonstrate the stability of the imposed ΔpH (pH_{in} 6.8/pH_{out} 8.0) well beyond the 5 min duration of *Step 1* in (**b**). Experimental procedures in (**e**) are identical to Supplementary Fig. 1.

SUPPLEMENTARY METHODS

Chemical synthesis of MsbA inhibitor G907

All reagents were obtained from commercial sources and used without further purification. Tetrahydrofuran was dried over sodium wire and distilled from a mixture of lithium aluminium hydride and calcium hydride with triphenylmethane as the indicator. Diethyl ether was distilled from calcium hydride and lithium aluminium hydride. Acetonitrile, dichloromethane, toluene and methanol were all distilled from calcium hydride. Petroleum ether was distilled before use and refers to the fraction with a boiling point of 40-60 °C.

Yields refer to chromatographically and spectroscopically pure compounds unless otherwise stated. Thin layer chromatography (TLC) was performed on commercially available glass Merck Kieselgel 60 F254 plates, and visualised UV irradiation (254 nm), or by staining with potassium permanganate dip. R_f values are quoted to the nearest 0.01. Flash column chromatography was performed using silica gel (Merck Kieselgel 60 F254, 230-400 mesh) under a positive pressure of nitrogen.

Proton magnetic resonance spectra were recorded using an internal deuterium lock (at 298 K unless stated otherwise) on Bruker DPX (400 MHz; 1H-13C DUL probe), Bruker Avance III HD (400 MHz; Smart probe), Bruker Avance III HD (500 MHz; Smart probe) and Bruker Avance III HD (500 MHz; DCH Cryoprobe) spectrometers. Assignments are supported by ^{1}H-^{1}H COSY, ^{1}H-^{13}C HSQC or ^{1}H-^{13}C HMBC spectra, or by analogy. Chemical shifts (δ_H) are quoted in ppm to the nearest 0.01 ppm and are referenced to the residual non-deuterated solvent peak: $CHCl_3$ (7.26) or d_5-DMSO (2.50). Discernible coupling constants for mutually coupled protons are reported as measured values in Hertz, rounded to the nearest 0.1 Hz. Data are reported as: chemical shift, number of nuclei, multiplicity (br, broad; s, singlet; d, doublet; t, triplet; q, quartet; m, multiplet; or a combination thereof), coupling constants and assignment. Two or more possible assignments were given when signals could not be distinguished by any means. Spectra were processed using TopSpin v. 4.0.1 (Bruker).

Carbon magnetic resonance spectra were recorded using an internal deuterium lock (at 298 K unless stated otherwise) on Bruker DPX (101 MHz), Bruker Avance III HD (101 MHz) and Bruker Avance III HD (126 MHz) spectrometers with broadband proton decoupling. Assignments are supported by DEPT editing, ^{1}H-^{13}C HSQC or ^{1}H-^{13}C HMBC spectra, or by analogy. Chemical shifts (δ_C) are quoted in ppm to the nearest 0.1 ppm and are referenced to the deuterated solvent peak: $CDCl_3$ (77.2) or d_6-DMSO (39.5). Data are reported as: chemical shift and assignment. An aryl (Ar), quaternary (C_Q), or two or more possible assignments were given when signals could not be distinguished by any means. Spectra were processed using TopSpin v. 4.0.1 (Bruker).

Infrared spectra were recorded neat on a Perkin Elmer Spectrum One FT-IR spectrometer with internal referencing. Selected absorption maxima (ν_{max}) are quoted in wavenumbers (cm^{-1}) and are assigned as: weak (w), medium (m), strong (s), broad (br), or a combination thereof. Melting points were obtained on a Büchi B-545 melting point apparatus and are uncorrected. High resolution mass spectrometry (HRMS) measurements were carried out on a Waters LCT Premier Time of Flight mass spectrometer or a Micromass Quadrupole Time of Flight mass spectrometer.

LCMS chromatographs were recorded on an Agilent 1200 series LC using a Supelcosil ABZ+PLUS column (33 mm x 4.6 mm, 3 μm), together with an ESCi Multi-Mode Ionisation Waters ZQ spectrometer using Masslynx 4.1 software. Samples are eluted using the following conditions: solvent A: 10 mM ammonium acetate + 0.1% formic acid in water; solvent B 95% acetonitrile + 5% water + 0.05% formic aid; linear gradient 0.0-0.7 minutes, 0-100% B from 0.7-4.2 min, 100% B from 4.2-7.7 min, 100-0% B from 7.7-8.5 min; flow rate 1 mL min^{-1}; injection volume: 10 μL.

G907 was prepared following the procedure reported by Ho *et al.*[2] The overall scheme is outlined below.

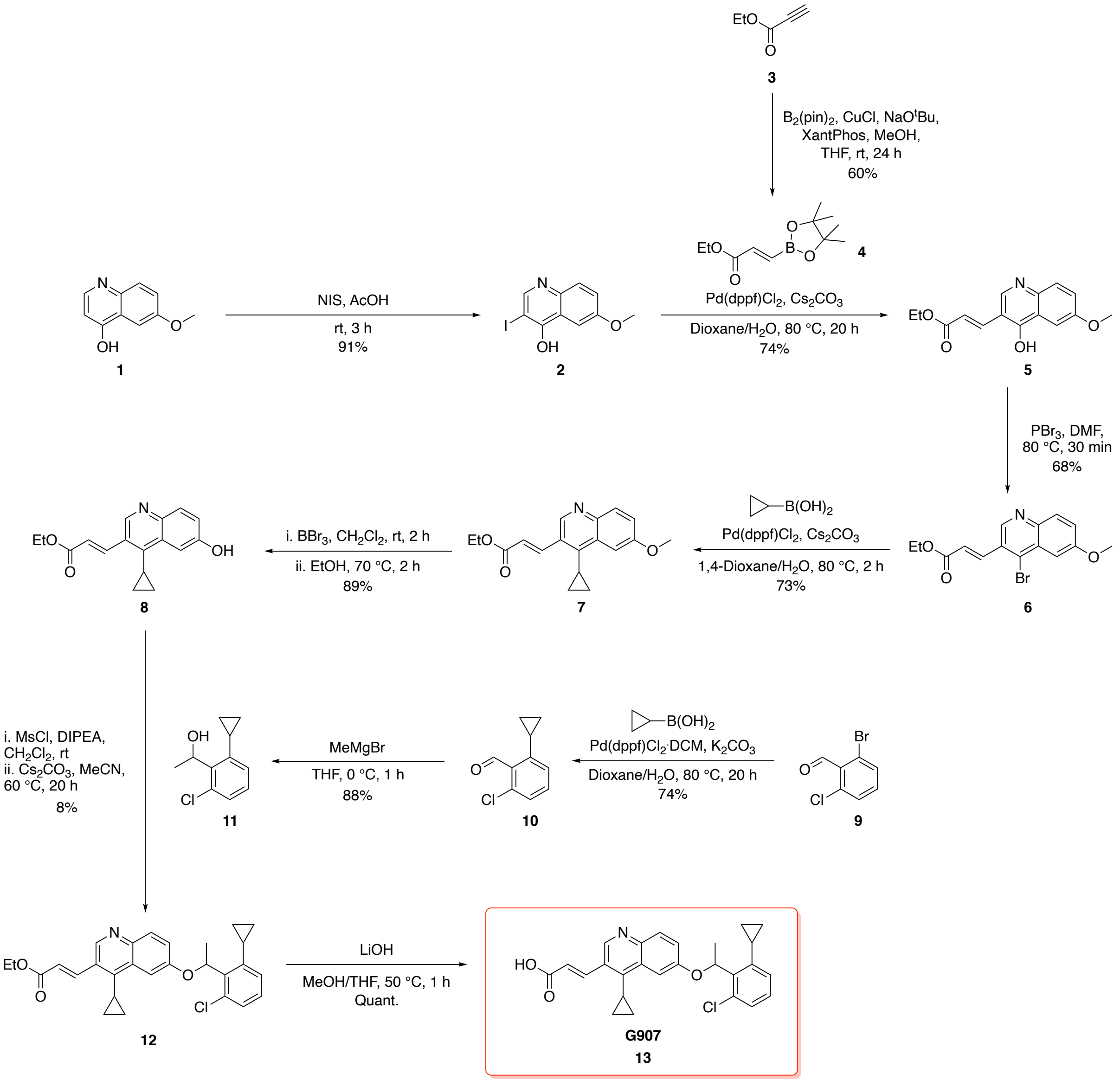

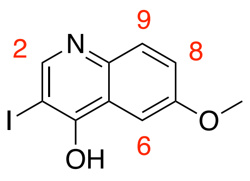

2

2 N-Iodosuccinimide (1.28 g, 5.71 mmol, 1 eq.) was added to a solution of **1** (1.00 g, 5.71 mmol) in acetic acid (12.5 mL). The reaction mixture was stirred at rt for 3 h and then the formed precipitate was filtered, washed with 40-60 petroleum ether (20 mL) and ethyl acetate (20 mL) to afford **2** as a grey solid (1.56 g, 5.18 mmol, 91%) which was carried on without further purification.

R_f: 0.70 (EtOAc); **IR:** (ν_{max}, cm^{-1}) = 3195 (w), 3158 (w), 3110 (w), 3082 (w), 3044 (w), 3006 (m), 2954 (m), 2900 (m), 2864 (w), 2173 (w) 1608 (w), 1576 (s), 1553 (s), 1499 (s), 1487 (s);

^{1}H NMR (400 MHz, d_6-DMSO): δ_H 12.19 (1H, d, J = 5.0 Hz, OH), 8.44 (1H, d, J = 5.8 Hz, H2), 7.55 (1H, d, J = 9.2 Hz, H9), 7.49 (1H, d, J = 2.3 Hz, H6), 7.32 (1H, dd, J = 8.9, 2.1 Hz, H8), 3.84 (1H, s, Me); **^{13}C NMR** (101 MHz, d_6-DMSO): δ_C 172.4 (C4), 156.1 (C7), 143.5 (C2), 134.1 (C10), 123.5 (C5), 122.4 (C8), 120.3 (C9), 104.6 (C6), 79.5 (C3), 55.4 (Me); **LCMS** (ESI+): m/z $[M + H]^+$ calculated for $C_{10}H_9INO_2$: 302.0; found 302.2.

Characterisation data is in accordance with the literature.[1]

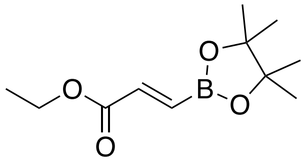

4

4 CuCl (31 mg, 0.31 mmol, 3 mol%), NaO^tBu (60 mg, 0.62 mmol, 6 mol%) and Xantphos ligand (179 mg, 0.31 mmol, 3 mol%) were placed in an oven-dried RBF and THF (10 mL) was added under nitrogen. The reaction mixture was stirred for at rt for 30 min and then, bis(pinacolato)diboron (2.89 g, 11.4 mmol, 1.1 eq.) and THF (5 mL) were added. The reaction mixture was stirred for another 10 min and then **3** (1.03 g, 10.4 mmol) was added, followed by MeOH (0.84 mL, 20.8 mmol, 2.0 eq.). The reaction mixture was stirred at rt for 24 h and then filtered through a pad of Celite and concentrated. The resulting residue was purified by flash column chromatography on silica eluting with EtOAc (0-20%) in 40-60 petroleum ether to yield the **4** as a colorless oil (1.41 g, 6.24 mmol, 60%).

R_f: 0.33 (5% EtOAc in 40-60 petroleum ether); **IR:** (ν_{max}, cm^{-1}) = 2981 (m), 1722 (s), 1627 (w), 1468 (w); **^{1}H NMR** (400 MHz, $CDCl_3$): δ_H 6.76 (1H, d, J = 18.2 Hz, C$\underline{H}$B$(pin)_2$), 6.62 (1H, d, J = 18.2 Hz, C$\underline{H}$$CO_2$Et), 4.20 (2H, q, J = 7.1 Hz, C$\underline{H}_2$$CH_3$), 1.29-1.26 (15H, m, CH_2C$\underline{H}_3$ and 4 x Me); **^{13}C NMR** (101 MHz, $CDCl_3$): δ_C 166.1 (CO_2Et), 138.9 (2 overlapping peaks, $\underline{C}$HB and $\underline{C}$HCO_2Et), 84.1 ($\underline{C}(CH_3)_2$), 60.7 (CH_2), 24.9 (4 x Me), 14.3 (Me); **HRMS** (ESI+): m/z $[M + H]^+$ calculated for $C_{11}H_{20}BO_4$: 227.1449; found 227.1451.

Characterisation data is in accordance with the literature.[1]

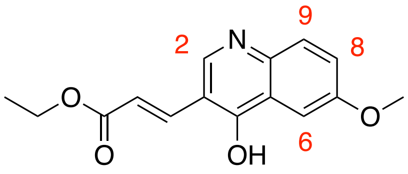

5

5 A mixture of **2** (1.77 g, 5.87 mmol), **4** (1.99 g, 8.80 mmol, 1.5 eq.), 1,1'-bis(diphenylphosphino)ferrocene palladium dichloride (300 mg, 0.41 mmol, 7 mol%) and caesium carbonate (5.74 g, 17.61 mmol, 3 eq.) in 1,4-dioxane (20 mL) and water (1 mL) was heated at 80 °C under a nitrogen atmosphere for 20 h and then concentrated under reduced pressure. The residue was dissolved in water (20 mL), stirred for another 1.5 h and filtered. The filter cake was then washed with methanol (50 mL) and ethyl acetate (30 mL) to afford **5** as a grey solid (1.18 g, 4.32 mmol, 74%) which was carried on without further purification.

R_f: 0.36 (EtOAc); **IR:** (ν_{max}, cm^{-1}) = 2897 (br m), 1899 (w), 1710 (m), 1633 (m), 1620 (m), 1581 (m), 1553 (m), 1519 (m); **^{1}H NMR** (400 MHz, d_6-DMSO): δ_H 12.45 (1H, br s, OH), 8.42 (1H, s, H2), 7.63 (1H, d, J = 15.7 Hz, CHCHCO_2Et), 7.58 (1H, d, J = 1.9 Hz, H6), 7.54 (1H, d, J = 8.9 Hz, H9), 7.32 (1H, dd, J = 8.6, 2.0 Hz, H8), 7.20 (1H, d, J = 15.7 Hz, CHCHCO_2Et), 4.15 (2H, q, J = 6.9 Hz, CH_2), 3.85 (3H, s, OMe), 1.25 (3H, t, J = 7.0 Hz, CH_3); **^{13}C NMR** (101 MHz, d_6-DMSO): δ_C 174.8 (COH), 167.6 (CO_2Et), 156.3 (COMe), 142.3 (C2), 140.9 (CHCHCO_2Et), 133.2 (C10), 126.9 (C5), 122.2 (C8), 120.5 (C9), 114.9 (CHCHCO_2Et), 112.9 (C3), 105.0 (C6), 59.4 (CH_2), 55.4 (OMe), 14.3 (Me); **LCMS** (ESI+): m/z $[M + H]^+$ calculated for $C_{15}H_{16}NO_4$: 274.1; found 274.3.

Characterisation data is in accordance with the literature.[1]

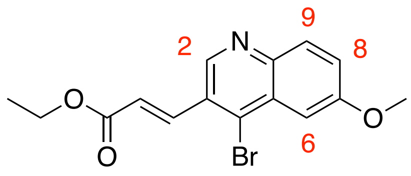

6

6 Phosphorus tribromide (480 µl, 5.14 mmol, 1.2 eq.) was added dropwise to a solution of **5** (1.17 g, 4.28 mmol) in N,N-dimethylformamide (10 mL) at rt. After addition, the mixture was stirred at 80 °C for 30 min and then cooled to 0 °C. The resulting mixture was quenched by addition of water (200 mL). The resulting solid was collected by filtration, washed with methanol (50 mL) and ethyl acetate (50 mL), and dried under reduced pressure to afford **6** as a grey solid (0.97 g, 2.89 mmol, 68%).

R_f: 0.65 (20% EtOAc in 40-60 petroleum ether); **IR:** (ν_{max}, cm^{-1}) = 3064 (w), 2978 (w), 2937 (w), 1850 (w), 1730 (s), 1628 (s), 1576 (w), 1554 (w); **^{1}H NMR** (400 MHz, d_6-DMSO): δ_H 9.10 (1H, s, H2), 8.05-8.00 (2H, m, CHCHCO_2Et and H9), 7.55-7.48 (2H, m, H6 and H8), 6.99 (1H, d, J = 16.0 Hz, CHCHCO_2Et), 4.25 (2H, q, J = 7.1 Hz, CH_2), 3.97 (3H, s, OMe), 1.29 (3H, t, J = 7.1 Hz, Me); **^{13}C NMR** (101 MHz, d_6-DMSO): δ_C 165.7 (CO_2Et), 159.2 (COMe), 146.0 (C2), 144.1 (CBr), 140.2 (CHCHCO_2Et), 134.1 (C3), 131.4 (C9), 128.1 (C10), 127.8 (C5), 123.7 (C8), 123.2 (CHCHCO_2Et), 105.4 (C6), 60.7 (CH_2), 55.8 (OMe), 14.2 (Me); **LCMS** (ESI+): m/z $[M + H]^+$ calculated for $C_{15}H_{15}{}^{81}BrNO_3$: 338.0; found 338.3.

Characterisation data is in accordance with the literature.[1]

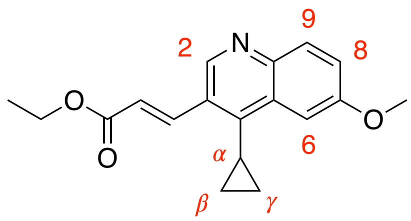

7

7 A mixture of **6** (938 mg, 2.79 mmol), caesium carbonate (1.82 g, 5.58 mmol, 2 eq.), cyclopropylboronicacid (479 mg, 5.58 mmol, 2 eq.) and 1,1'- bis(diphenylphosphino)ferrocene

palladium dichloride (102 mg, 0.14 mmol, 5 mol%) in 1,4-dioxane (16 mL) and water (4 mL) was heated at 80 °C under a nitrogen atmosphere for 2 h and concentrated under reduced pressure. The resulting residue was purified by flash column chromatography on silica eluting with EtOAc (0-20%) in 40-60 petroleum ether to yield **7** as a white solid (607 mg, 2.04 mmol, 73%).

R_f: 0.39 (20% EtOAc in 40-60 petroleum ether); **IR:** (ν_{max}, cm^{-1}) = 3073 (w), 2927 (w), 1713 (s), 1631 (s), 1508 (m); **^{1}H NMR** (400 MHz, d_6-DMSO): δ_H 9.02 (1H, s, H2), 8.39 (1H, d, J = 16.3 Hz, $C\underline{H}CHCO_2Et$), 7.92 (1H, d, J = 9.1 Hz, H9), 7.77 (1H, d, J = 2.8 Hz, H6), 7.43 (1H, dd, J = 9.1 , 2.8 Hz, H8), 6.78 (1H, d, J = 16.2 Hz, $CHC\underline{H}CO_2Et$), 4.24 (2H, q, J = 7.1 Hz, CH_2), 3.94 (3H, s, OMe), 2.23 (1H, m, Hα), 1.35-1.32 (2H, m, Hβ and Hγ), 1.29 (3H, t, J = 7.1 Hz, Me), 0.63-0.59 (2H, m, Hβ and Hγ); **^{13}C NMR** (101 MHz, d_6-DMSO): δ_C 166.1 (CO_2Et), 157.4 (COMe), 146.2 (C2), 145.4 (C4), 143.7 (C10), 140.4 ($\underline{C}HCHCO_2Et$), 131.0 (C9), 129.1 (C5), 127.2 (C3), 121.9 (C8), 120.1 ($CH\underline{C}HCO_2Et$), 104.1 (C6), 60.2 (CH_2), 55.4 (OMe), 14.2 (Me), 10.0 (Cα), 8.0 (Cβ and Cγ); **LCMS** (ESI+): m/z $[M + H]^+$ calculated for $C_{18}H_{20}NO_3$: 298.1; found 298.4.

Characterisation data is in accordance with the literature.[1]

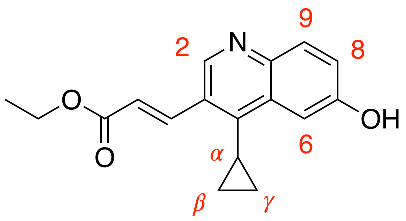

8

8 Tribromoborane (1 M in CH_2Cl_2, 10.1 mL, 10.11 mmol, 5 eq.) was added dropwise to a solution of **7** (601 mg, 2.02 mmol) in CH_2Cl_2 (20 mL) at -78 °C. The mixture was stirred at rt for 2 h and then quenched by addition of ethanol (20 mL) at -78 °C. The resulting mixture was stirred at 70 °C for 2 h and then concentrated under reduced pressure. The resulting residue was recrystallised from ethanol and dried to afford **8** as an off-white solid (511 mg, 1.80 mmol, 89%).

R_f: 0.23 (20% EtOAc in 40-60 petroleum ether); m.p.: 226-228 °C; **IR:** (ν_{max}, cm^{-1}) = 3094 (m, br), 2984 (w), 2892 (w), 2793 (w), 1713 (s), 1632 (s), 1612 (m), 1580 (m), 1551 (m), 1504 (m); **^{1}H NMR** (400 MHz, d_6-DMSO): δ_H 10.85 (1H, br s, OH), 9.31 (1H, s, H2), 8.31 (1H, d, J = 16.2 Hz, $C\underline{H}CHCO_2Et$), 8.07 (1H, d, J = 9.1 Hz, H9), 7.90 (1H, d, J = 2.5 Hz, H6), 7.60 (1H, dd, J = 9.1, 2.5 Hz, H8), 6.93 (1H, d, J = 16.2 Hz, $CHC\underline{H}CO_2Et$), 4.26 (2H, t, J = 7.1 Hz, CH_2), 2.46-2.39 (1H, m, Hα), 1.41-1.36 (2H, m, Hβ and Hγ), 1.30 (3H, t, 7.1 Hz, Me), 0.70-0.66 (2H, m, Hβ and Hγ); **^{13}C NMR** (101 MHz, d_6-DMSO): δ_C 165.7 (CO_2Et), 157.7 (COH), 152.6 (C4), 141.2 (C2), 138.3 ($\underline{C}HCHCO_2Et$), 134.2 (C10), 130.8 (C5), 128.3 (C3), 125.6 (C8), 125.1 (C9), 122.6 ($CH\underline{C}HCO_2Et$), 107.6 (C6), 60.6 (CH_2), 14.2 (Me), 11.2 (Cα), 8.4 (Cβ and Cγ); **LCMS** (ESI+): m/z $[M + H]^+$ calculated for $C_{17}H_{18}NO_3$: 284.1; found 284.4.

Characterisation data is in accordance with the literature.[1]

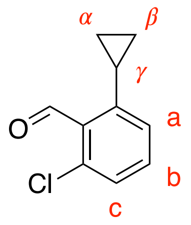

10

10 A mixture of **9** (2.21 g, 10.07 mmol), cyclopropylboronic acid (0.86 g, 10.07 mmol, 1 eq.), Pd(dppf)Cl_2 (368 mg, 0.50 mmol, 5 mol%) and potassium carbonate (4.18 g, 30.21 mmol, 3 eq.) in a mixture of 1,4-dioxane/water (20 mL, 4:1) was heated under N_2 at 80 °C for 20 h. The resulting mixture was cooled, diluted with EtOAc and filtered through Celite. The filtrate was washed with water (30 mL) and brine (30 mL), dried over sodium sulfate and concentrated. The resulting residue was purified by flash column chromatography on silica eluting with EtOAc (0-10%) in 40-60 petroleum ether to yield **10** as a yellow oil (1.35 g, 7.47 mmol, 74%).

R_f: 0.46 (5% EtOAc in 40-60 petroleum ether); **IR:** (ν_{max}, cm^{-1}) = 2936 (w), 2343 (w), 2204 (w), 2091 (w), 1734 (m), 1593 (w), 1569 (w); **^{1}H NMR** (400 MHz, $CDCl_3$): δ_H 10.70 (1H, d, J = 0.5 Hz, CHO), 7.33 (1H, t, J = 7.8 Hz, Hc), 7.25 (1H, dd, J = 8.0, 1.1 Hz, Hb), 6.98-6.96 (1H, d, J = 7.8 Hz, Ha), 2.75-2.68 (1H, m, Hγ), 1.07-1.02 (2H, m, Hα and Hβ), 0.71-0.67 (2H, m, Hα and Hβ); **^{13}C NMR** (101 MHz, $CDCl_3$): δ_C 192.8 (CHO), 147.8 (CCl), 138.0 (CCγ), 133.6 (Cc), 132.3 (CCHO), 128.0 (Cb), 124.8 (Ca), 13.0 (Cγ), 9.4 (2 overlapping peaks, Cα and Cβ); **HRMS** (ESI+): m/z $[M + H]^+$ calculated for $C_{10}H_{10}{}^{35}ClO$: 181.0420; found 181.0414.

Characterisation data is in accordance with the literature.[1]

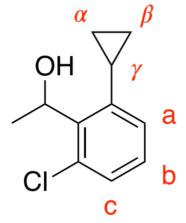

11

11 Methylmagnesium bromide (4.25 mL of 3 M solution in ether, 12.74 mmol, 2 eq.) was added to an ice-cold solution of **10** (1.15 g, 6.37 mmol) in THF (20 mL) and stirred for 1 h at room temperature. The reaction mixture was quenched with saturated aqueous ammonium chloride solution (20 mL) before being extracted with ethyl acetate (3 x 30 mL). The organic layer was washed with water (30 mL), brine (30 mL), dried over sodium sulfate, and concentrated to afford **11** as a white solid (1.11 g, 5.63 mmol, 88%) which was carried on without further purification.

R_f: 0.67 (20% EtOAc in 40-60 petroleum ether); **IR:** (ν_{max}, cm^{-1}) = 3262 (br m), 3081 (w), 2977 (w), 2933 (w), 2868 (w), 1591 (m), 1567 (m); **^{1}H NMR** (400 MHz, d_6-DMSO): δ_H 7.16 (1H, d, J = 7.9 Hz, Hc), 7.10 (1H, t, J = 7.8 Hz, Hb), 6.82 (1H, d, J = 7.6 Hz, Ha), 5.62-5.56 (1H, m,

CHOH), 5.35 (1H, d, J = 2.8 Hz, OH), 2.84-2.76 (1H, m, Hγ), 1.49 (3H, d, J = 6.8 Hz, Me), 1.00-0.89 (2H, m, Hα and Hβ) , 0.87-0.81 (1H, m, Hα or Hβ), 0.55-0.49 (1H, m, Hα or Hβ); **^{13}C NMR** (101 MHz, d_6-DMSO): δ_C 144.4 (CCγ), 140.9 (CCOH), 131.8 (CCl), 127.9 (Cb), 126.7 (Cc), 123.3 (Ca), 65.7 (COH), 22.0 (Me), 12.4 (Cγ), 10.5 (Cα or Cβ), 8.2 (Cα or Cβ); **HRMS** (ESI+): m/z $[M + H]^+$ calculated for $C_{11}H_{14}{}^{35}ClO$: 197.0728; found 197.0723.

Characterisation data is in accordance with the literature.[1]

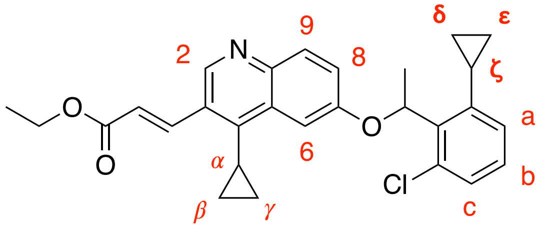

12

12 **11** (432 mg, 2.20 mmol) was dissolved in CH_2Cl_2 (5 mL), DIPEA (765 µl, 4.39 mmol, 2 eq.) was added, and the reaction mixture was cooled to 0 °C. Methanesulfonyl chloride (255 µl, 3.29 mmol, 1.5 eq.) was added and the reaction allowed to warm to room temperature. The reaction mixture was diluted with CH_2Cl_2 (5 mL) and then washed with saturated aqueous sodium bicarbonate (10 mL), water (10 mL), and brine (10 mL). The organic layer was the dried over sodium sulfate, filtered, and concentrated. The residue appeared to be unstable so was used immediately in the next step. **8** (178 mg, 0.63 mmol) and Cs_2CO_3 (205 mg, 0.63 mmol, 1 eq.) were combined in acetonitrile (10 mL) and stirred for 15 min at room temperature. The methanesulfonate (604 mg, 2.20 mmol, 3.5 eq.) was added and the reaction mixture heated at 60 °C for 20 h. The reaction was allowed to cool to rt, diluted with EtOAc (20 mL), washed with water (20 mL) and brine (20 mL), and then dried (Na_2SO_4), filtered, and concentrated. The resulting residue was purified by flash column chromatography on silica eluting with EtOAc (0-30%) in 40-60 petroleum ether to yield **12** as a white solid (21 mg, 0.05 mmol, 8%).

R_f: 0.41 (20% EtOAc in 40-60 petroleum ether); **IR:** (ν_{max}, cm^{-1}) = 2980 (w), 1711 (s), 1626 (s), 1505 (m), 1435 (m); **^{1}H NMR** (400 MHz, d_6-DMSO): δ_H 8.97 (1H, s, H2), 8.31 (1H, d, J = 16.2 Hz, CHCHCO$_2$Et), 7.89 (1H, d, J = 9.0 Hz, H9), 7.49 (1H, d, J = 2.4 Hz, H6), 7.45 (1H, dd, J = 9.0, 2.4 Hz, H8), 7.30 (1H, d, J = 7.9 Hz, Ha/Hc), 7.17 (1H, t, J = 7.9 Hz, Hb), 6.83 (1H, d, J = 7.6 Hz, Ha/Hc), 6.73 (1H, d, J = 16.2 Hz, CHCHCO$_2$Et), 6.28 (1H, q, J = 6.6 Hz, OCHCH$_3$), 4.21 (2H, q, J = 7.1 Hz, CH$_2$), 2.68-2.58 (1H, m, Hζ), 2.08-2.01 (1H, m, Hα), 1.83 (3H, d, J = 6.6 Hz, OCHCH$_3$), 1.27 (3H, t, J = 7.1 Hz, Me), 1.23-1.20 (1H, m, Hβ or Hγ), 1.08-1.05 (1H, m, Hβ or Hγ), 1.03-1.00 (1H, m, Hδ or Hε), 0.93-0.86 (1H, m, Hδ or Hε), 0.71-0.67 (1H, m, Hδ or Hε), 0.55-0.50 (1H, m, Hδ or Hε), 0.49-0.44 (1H, m, Hβ or Hγ), 0.10-0.03 (1H, m, Hβ or Hγ); **^{13}C NMR** (101 MHz, d_6-DMSO): δ_C 166.1 (CO$_2$Et), 155.1 (C7), 146.4 (C2), 145.3 (CCα), 143.7 (CCζ), 140.5 (CHCHCO$_2$Et), 136.0 (CCHCH$_3$), 132.4 (CCl), 131.1 (C9), 129.2 (Cb), 128.8 (C10), 127.4 (Ca/Cc), 124.1 (2 overlapping peaks, Ca/Cc, and C5), 123.1 (C8), 120.2 (CHCHCO$_2$Et), 105.6 (C6), 73.3 (OCHCH$_3$), 60.3 (CH$_2$), 20.5 (OCHCH$_3$), 14.2 (Me), 12.6 (Cζ), 9.9 (Cα), 9.2 (2 overlapping peaks, Cδ and Cε), 8.1 (Cβ or Cγ), 7.6 (Cβ or Cγ); **LCMS** (ESI+): m/z $[M + H]^+$ calculated for $C_{28}H_{29}{}^{35}ClNO_3$: 462.2; found 462.4.

Characterisation data is in accordance with the literature.[1]

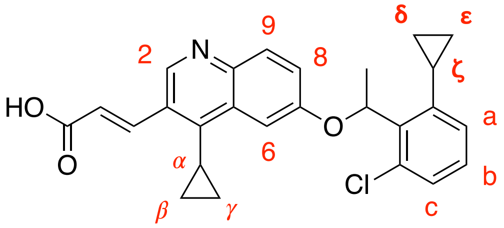

13

G907 (13) **12** (11 mg, 0.024 mmol) was dissolved in MeOH/THF (1 mL, 1:1). 1 M LiOH solution (0.5 mL) was added and the mixture stirred at 50 °C for 1.5 h. The reaction mixture was then cooled to room temperature, acidified with 1 M HCl solution, and extracted with EtOAc (3 x 5 mL). The organic layers were washed with water (5 mL) and brine (5 mL), dried over Na_2SO_4, filtered, and concentrated to afford G907 (**13**) as a white solid with no further purification (10 mg, 0.024 mmol, Quant.).

R_f: 0.08 (EtOAc); **IR:** (ν_{max}, cm^{-1}) = 3390 (w), 3002 (w), 2494 (w), 2358 (w), 2162 (w), 1976 (s), 1697 (m), 1616 (m), 1504 (m), 1436 (m); **^{1}H NMR** (400 MHz, d_6-DMSO): δ_H 12.59 (1H, s, CO_2H), 8.93 (1H, s, H2), 8.24 (1H, d, J = 16.2 Hz, C<u>H</u>CHCO$_2$Et), 7.88 (1H, d, J = 9.0 Hz, H9), 7.48 (1H, d, J = 2.3 Hz, H6), 7.44 (1H, dd, J = 9.1, 2.6 Hz, H8), 7.30 (1H, d, J = 7.9 Hz, Ha/Hc), 7.17 (1H, t, J = 7.9 Hz, Hb), 6.83 (1H, d, J = 7.5 Hz, Ha/Hc), 6.64 (1H, d, J = 16.2 Hz, CHC<u>H</u>CO$_2$Et), 6.27 (1H, q, J = 6.6 Hz, OC<u>H</u>CH$_3$), 2.66-2.59 (1H, m, Hζ), 2.06-1.99 (1H, m, Hα), 1.83 (3H, d, J = 6.6 Hz, OCHC<u>H</u>$_3$), 1.24-1.19 (1H, m, Hβ or Hγ), 1.08-1.04 (1H, m, Hβ or Hγ), 1.03-0.98 (1H, m, Hδ or Hε), 0.93-0.86 (1H, m, Hδ or Hε), 0.70-0.67 (1H, m, Hδ or Hε), 0.55-0.50 (1H, m, Hδ or Hε), 0.48-0.45 (1H, m, Hβ or Hγ), 0.07-0.04 (1H, m, Hβ or Hγ); **^{13}C NMR** (101 MHz, d_6-DMSO): δ_C 167.3 (CO_2H), 155.1 (C7), 146.3 (C2), 145.0 (<u>C</u>Cα), 143.5 (<u>C</u>Cζ), 139.8 (<u>C</u>HCHCO$_2$Et), 135.8 (<u>C</u>CHCH$_3$), 132.3 (CCl), 131.0 (C9), 129.2 (Cb), 128.8 (C10), 127.6 (Ca/Cc), 124.1 (2 overlapping peaks, Ca/Cc, and C5), 122.9 (C8), 121.4 (CH<u>C</u>HCO$_2$Et), 105.5 (C6), 73.3 (O<u>C</u>HCH$_3$), 20.4 (OCH<u>C</u>H$_3$), 12.5 (Cζ), 9.9 (Cα), 9.2 (2 overlapping peaks, Cδ and Cε), 8.1 (Cβ or Cγ), 7.6 (Cβ or Cγ); **LCMS**(ESI+): m/z $[M + H]^+$ calculated for $C_{26}H_{25}{}^{35}ClNO_3$: 434.2; found 434.7.

Characterisation data is in accordance with the literature.[1]

^{1}H and ^{13}C NMR Spectra

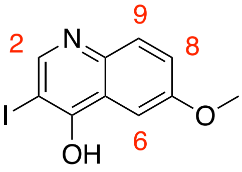

2

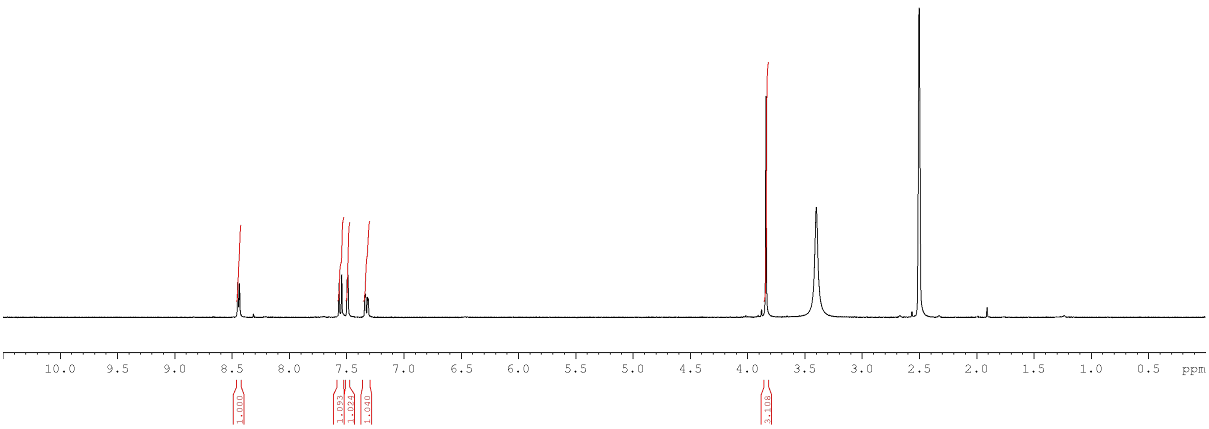

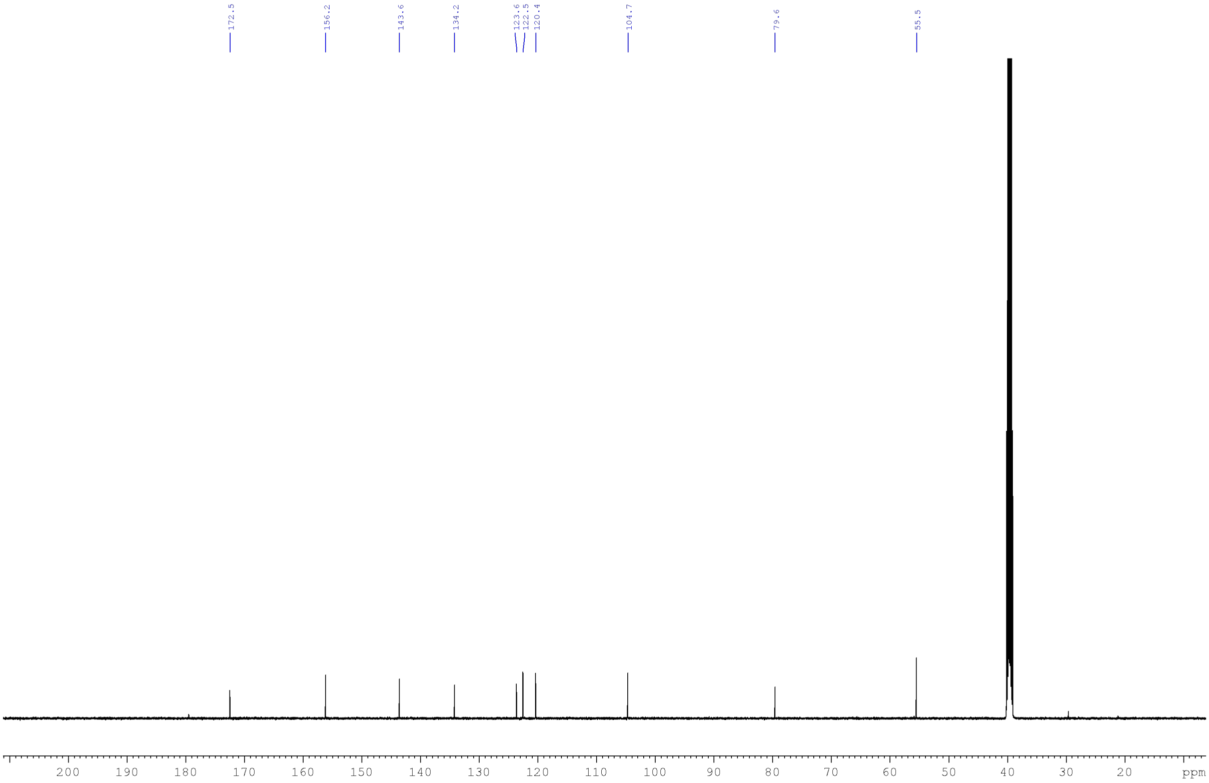

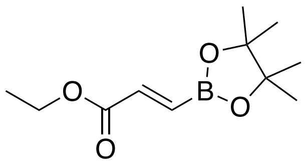
O
B
O
O
O

4

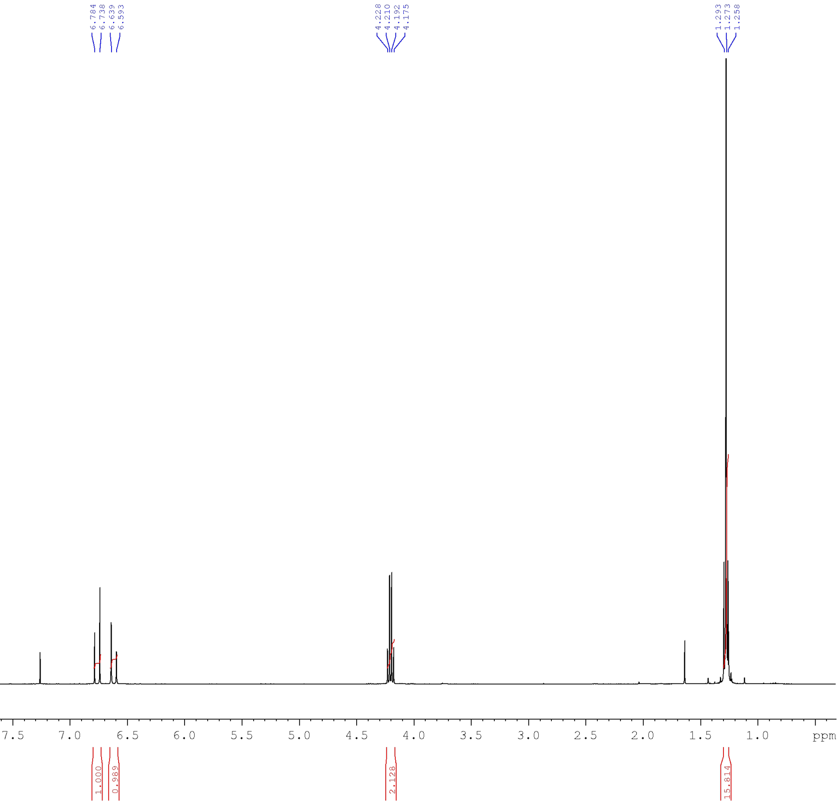
6.784
6.738
6.639
6.593
4.228
4.210
4.192
4.175
1.293
1.273
1.258
1.000
0.989
2.128
15.814
ppm

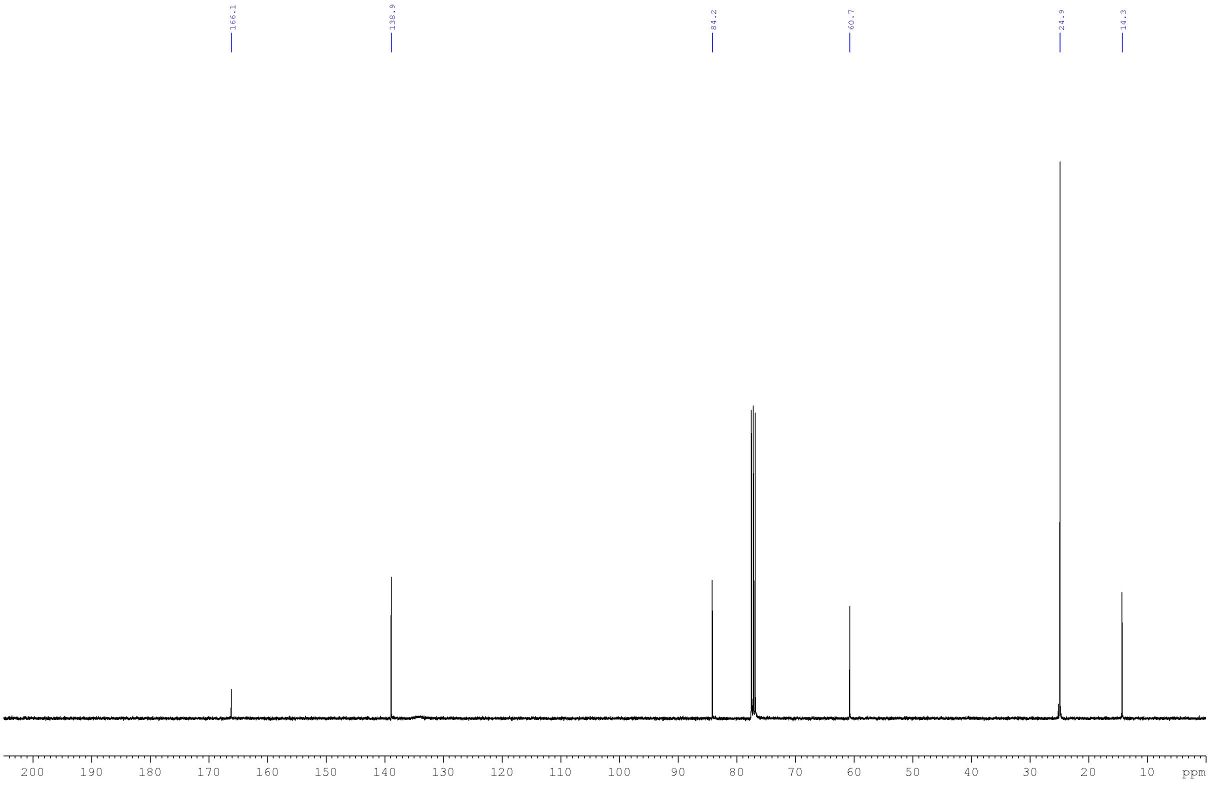
166.1
138.9
84.2
60.7
24.9
14.3
ppm

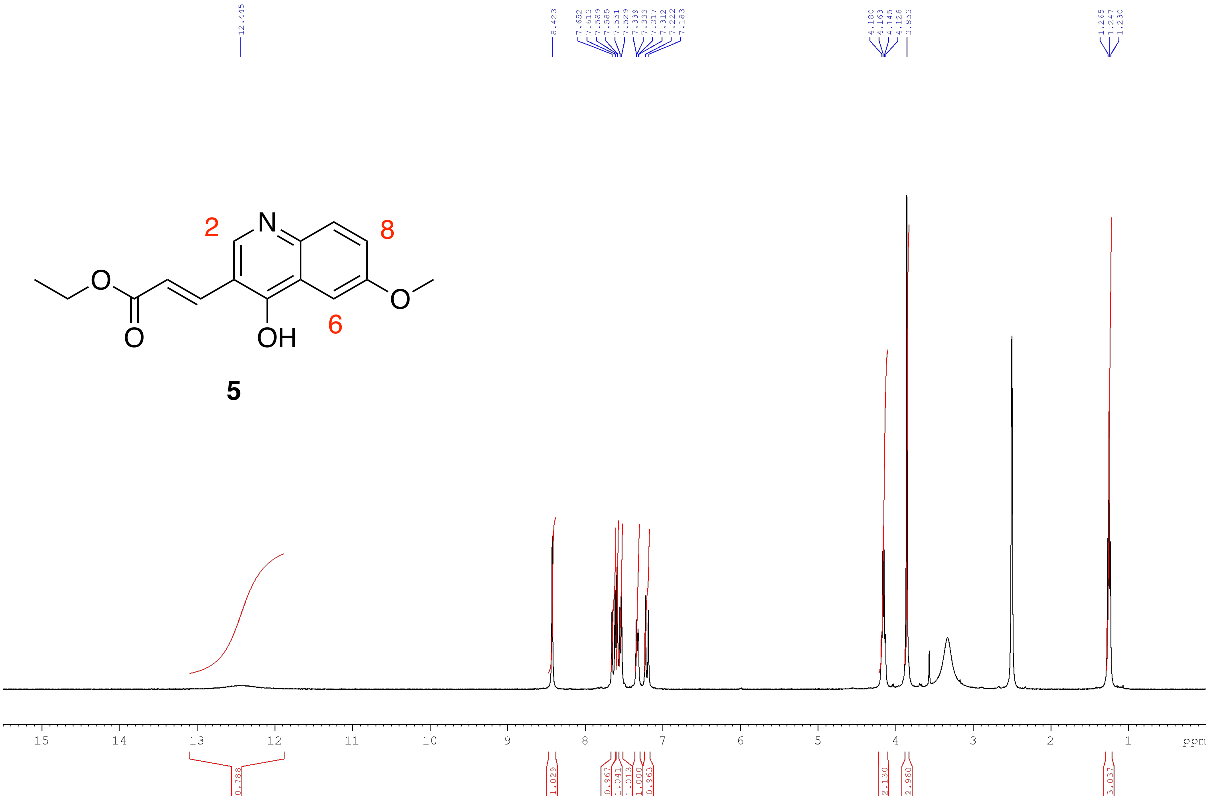
2
N
8
O
O
OH
6
O
5
ppm

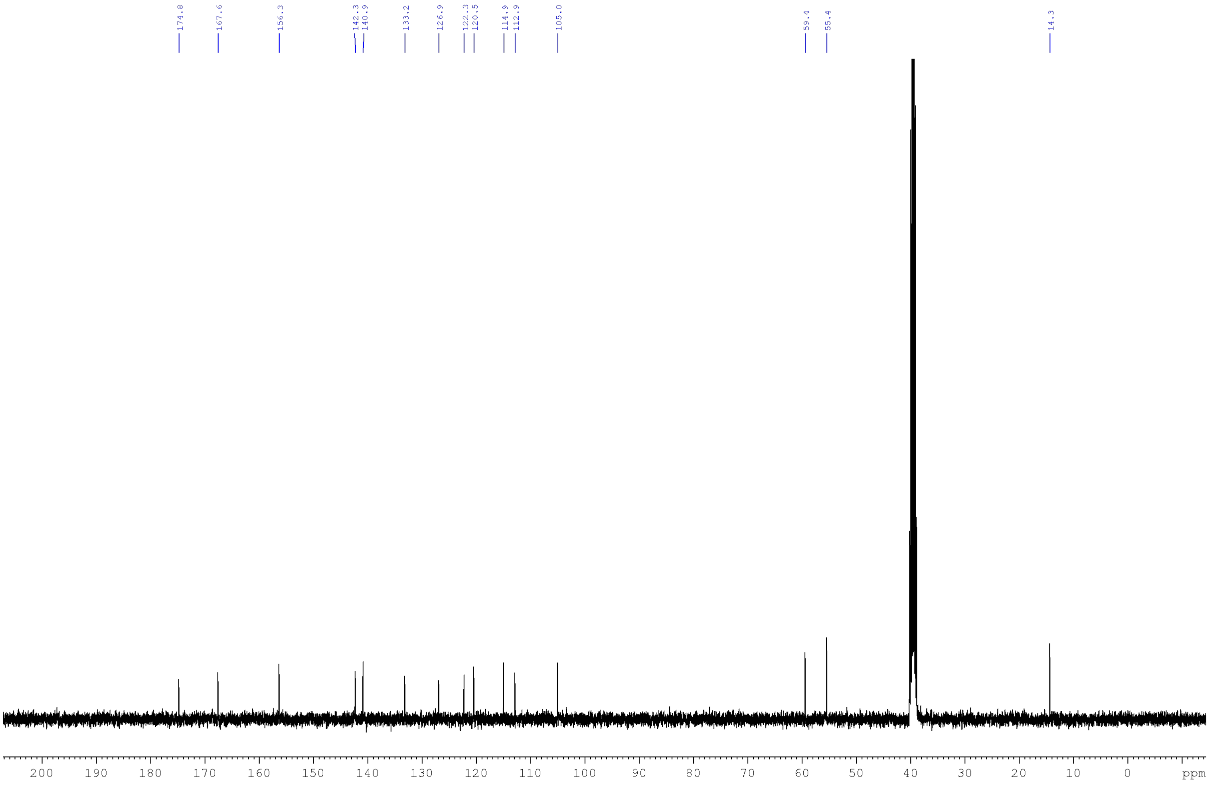
174.8
167.6
156.3
142.3
140.9
133.2
126.9
122.3
120.5
114.9
112.9
105.0
59.4
55.4
14.3
ppm

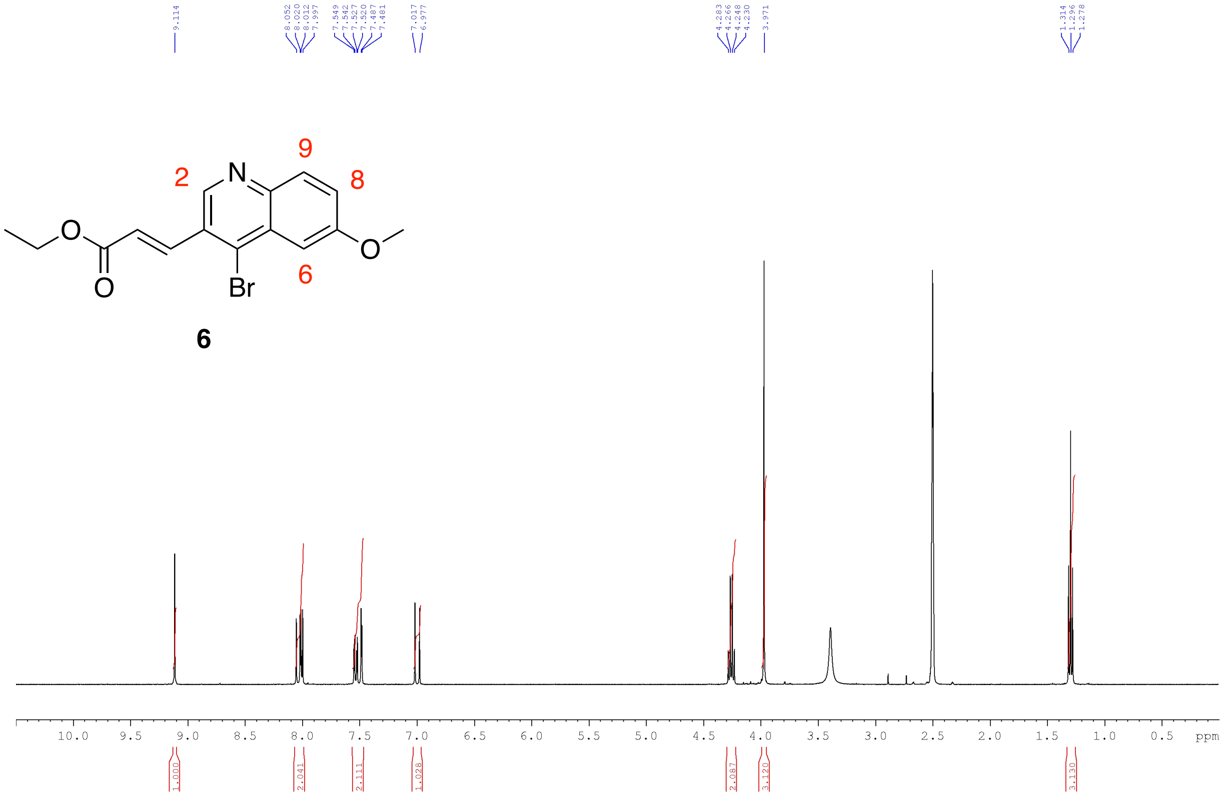
2
N
9
8
O
O
O
Br
6
9.114
8.052 8.020 8.012 7.997
7.549 7.542 7.527 7.520 7.487 7.481
7.017 6.977
4.283 4.266 4.248 4.230
3.971
1.314 1.296 1.278
10.0 9.5 9.0 8.5 8.0 7.5 7.0 6.5 6.0 5.5 5.0 4.5 4.0 3.5 3.0 2.5 2.0 1.5 1.0 0.5 ppm
1.000
2.041
2.111
1.028
2.087
3.120
3.130

6

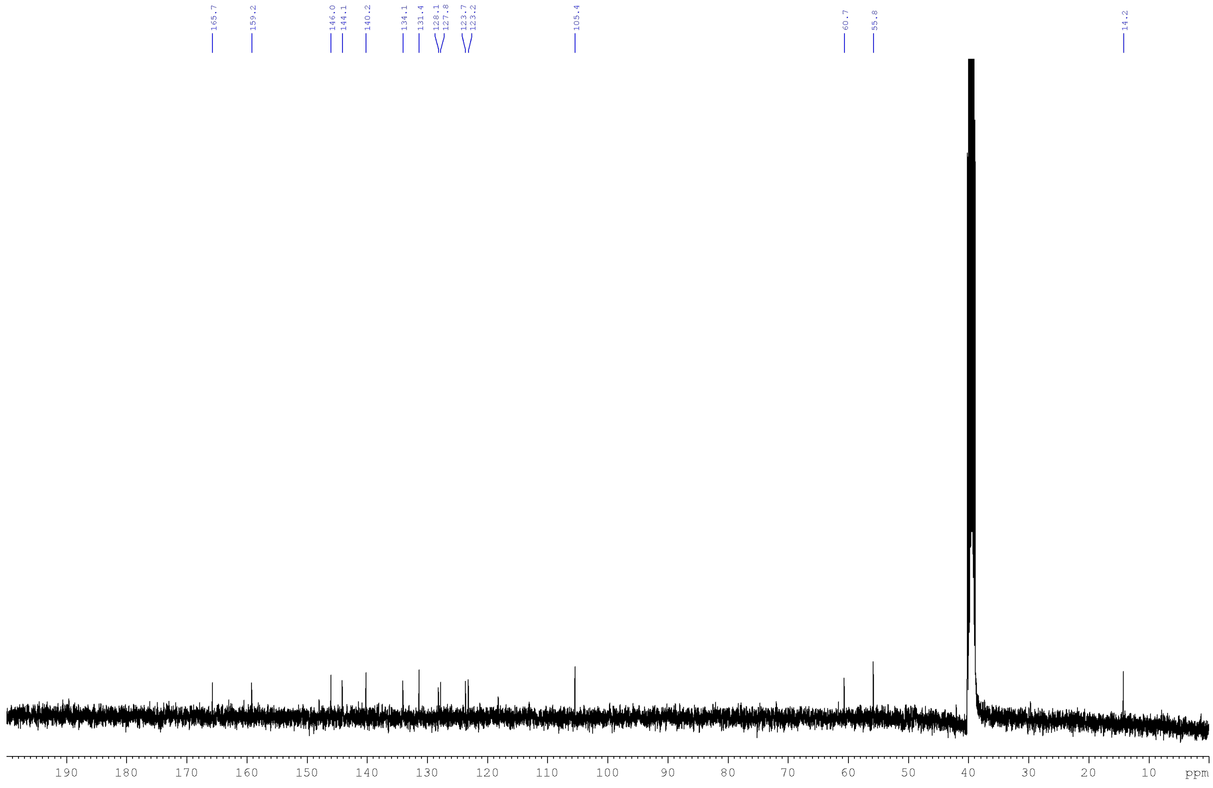
165.7
159.2
146.0 144.1
140.2
134.1 131.4 128.1 127.8 123.7 123.2
105.4
60.7
55.8
14.2
190 180 170 160 150 140 130 120 110 100 90 80 70 60 50 40 30 20 10 ppm

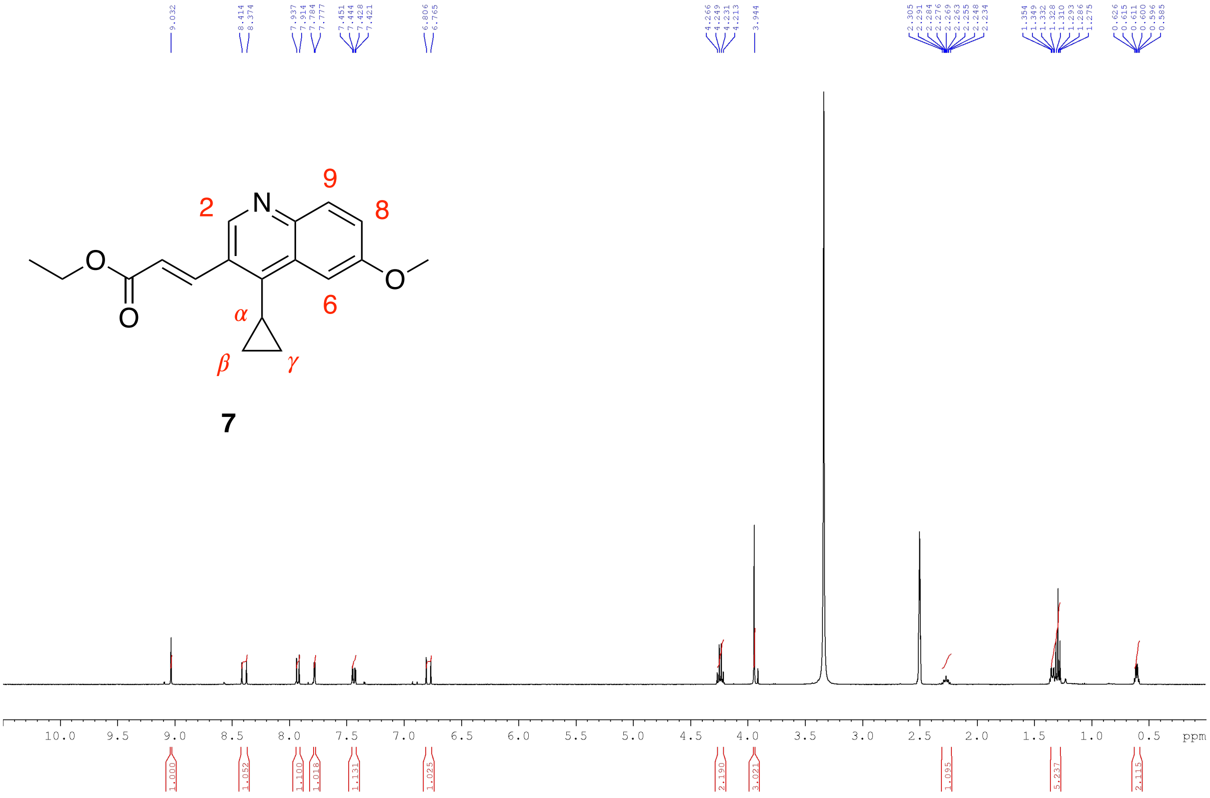
9.032
8.414
8.374
7.937
7.914
7.784
7.777
7.451
7.444
7.428
7.421
6.806
6.765
4.266
4.249
4.231
4.213
3.944
2.305
2.291
2.284
2.276
2.269
2.263
2.255
2.248
2.234
1.354
1.349
1.332
1.328
1.310
1.293
1.286
1.275
0.626
0.615
0.611
0.600
0.596
0.585
2
N
9
8
O
O
O
6
α
β
γ
7
10.0 9.5 9.0 8.5 8.0 7.5 7.0 6.5 6.0 5.5 5.0 4.5 4.0 3.5 3.0 2.5 2.0 1.5 1.0 0.5 ppm
1.000
1.052
1.100
1.018
1.131
1.025
2.190
3.021
1.095
5.237
2.115

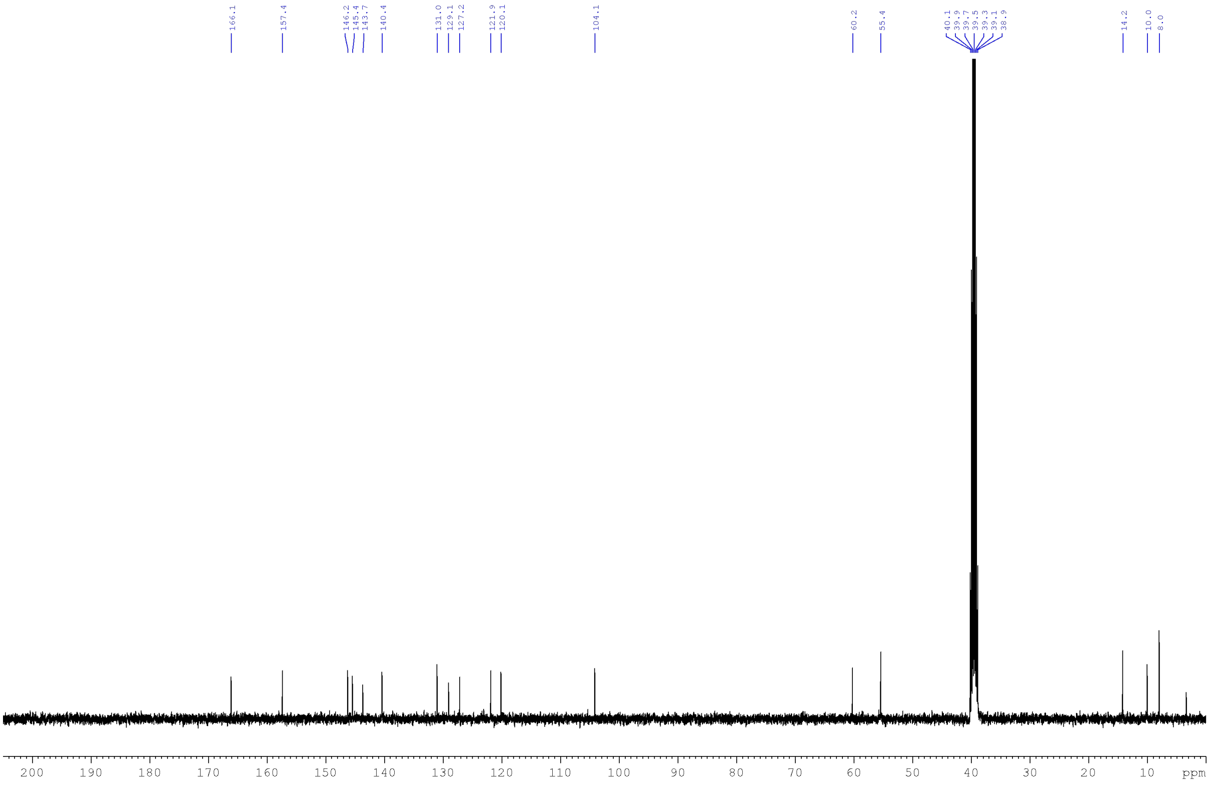
166.1
157.4
146.2
145.4
143.7
140.4
131.0
129.1
127.2
121.9
120.1
104.1
60.2
55.4
40.1
39.9
39.7
39.5
39.3
39.1
38.9
14.2
10.0
8.0
200 190 180 170 160 150 140 130 120 110 100 90 80 70 60 50 40 30 20 10 ppm

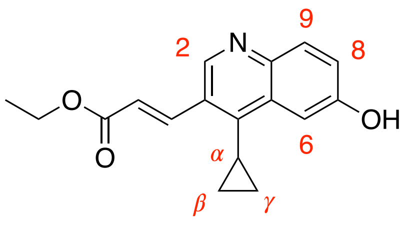

2
N
9
8
O
O
OH
6
α
β
γ

8

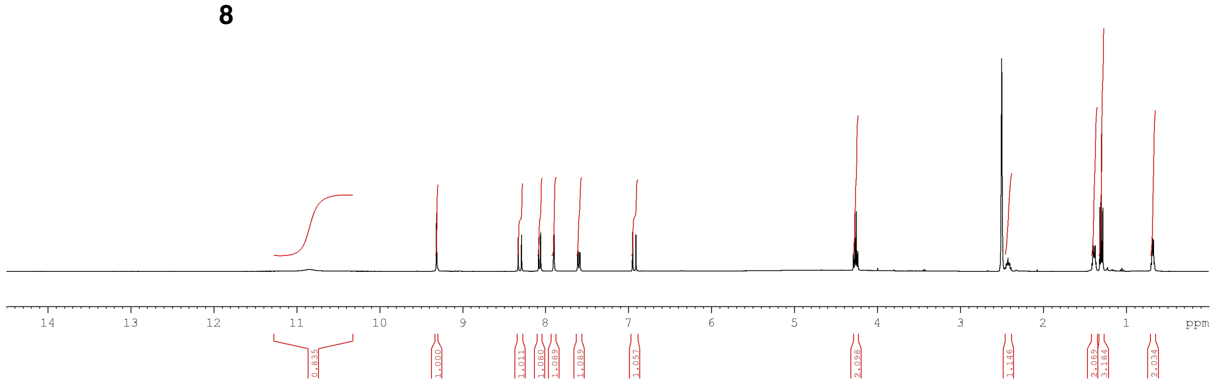

10.847
9.313
8.329
8.288
8.081
8.058
7.902
7.895
7.612
7.606
7.590
7.583
6.950
6.909
4.287
4.269
4.252
4.234
2.460
2.445
2.439
2.424
2.409
2.402
2.387
1.410
1.398
1.394
1.316
1.299
1.281
0.698
0.683
0.672
0.669
0.658
14 13 12 11 10 9 8 7 6 5 4 3 2 1 ppm
0.835
1.000
1.011
1.000
1.089
1.089
1.057
2.098
1.146
2.069
3.184
2.034

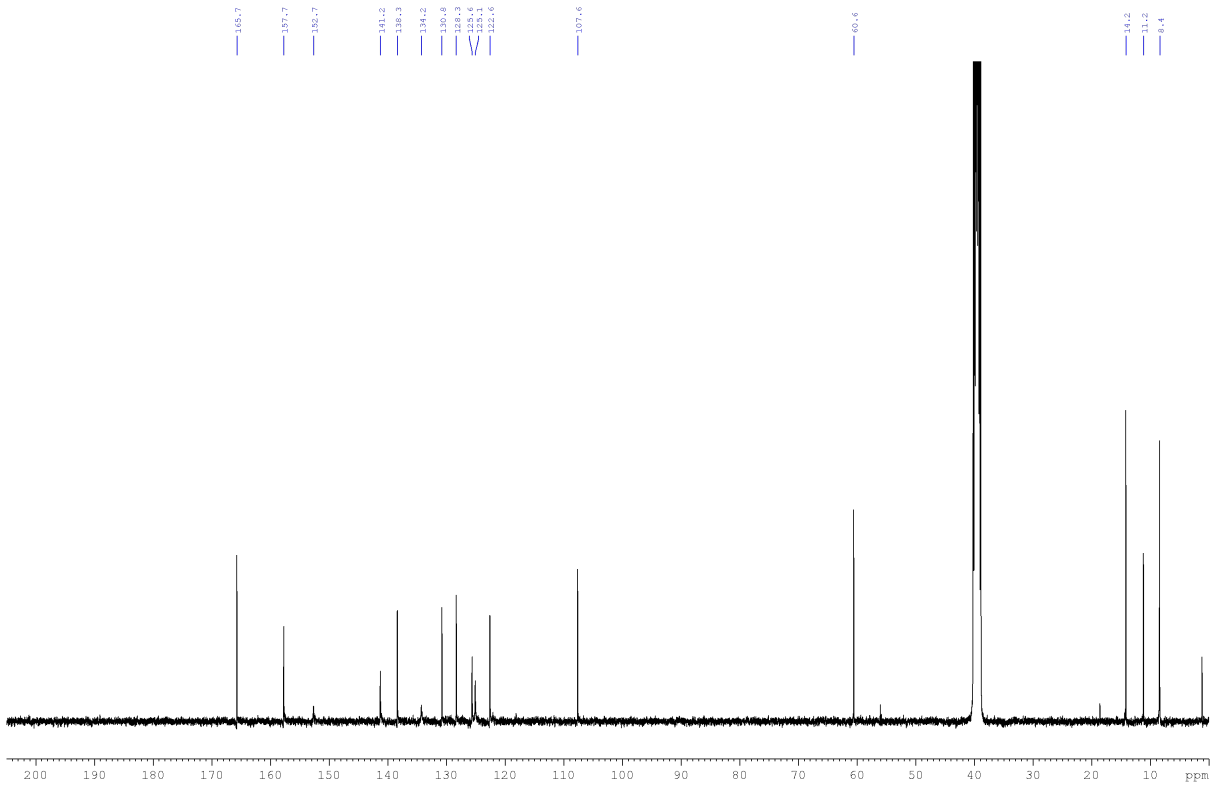

165.7
157.7
152.7
141.2
138.3
134.2
130.8
128.3
125.6
125.1
122.6
107.6
60.6
14.2
11.2
8.4
200 190 180 170 160 150 140 130 120 110 100 90 80 70 60 50 40 30 20 10 ppm

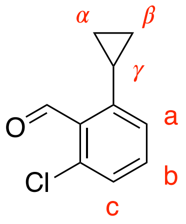
α
β
γ
a
b
c
O
Cl

10

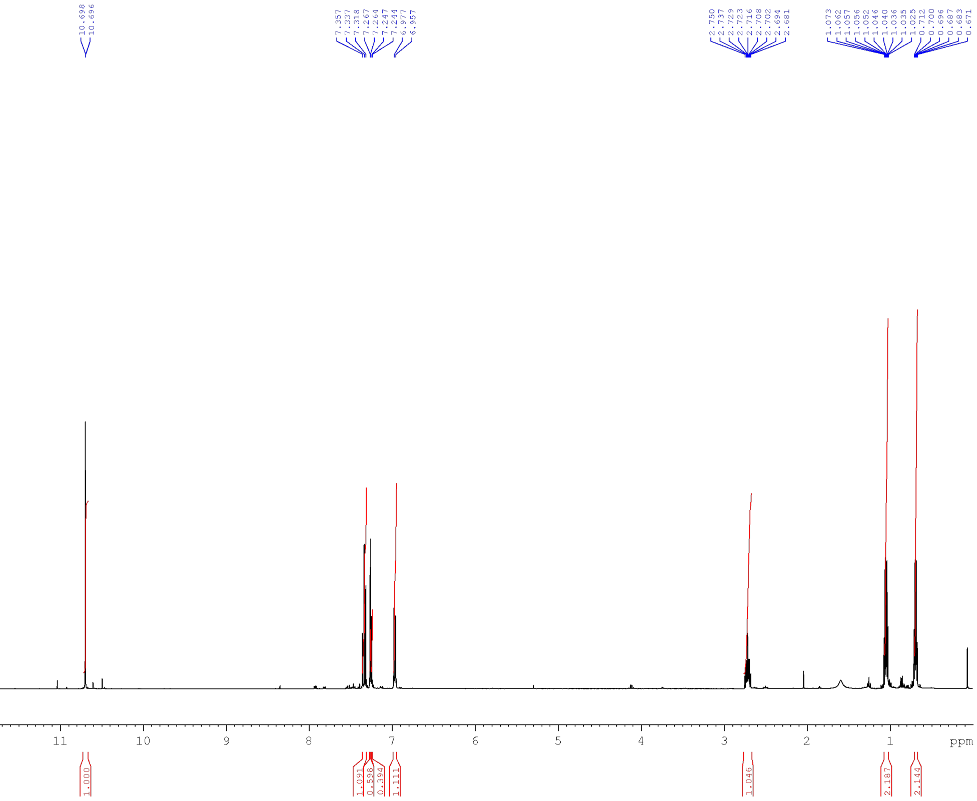
10.698
10.696
7.357
7.337
7.318
7.267
7.264
7.247
7.244
6.977
6.957
2.750
2.737
2.729
2.723
2.716
2.708
2.702
2.694
2.681
1.073
1.062
1.057
1.056
1.052
1.046
1.040
1.036
1.035
1.025
0.712
0.700
0.696
0.687
0.683
0.671
14
13
12
11
10
9
8
7
6
5
4
3
2
1
ppm
1.000
1.091
0.598
0.394
1.111
1.046
2.187
2.144

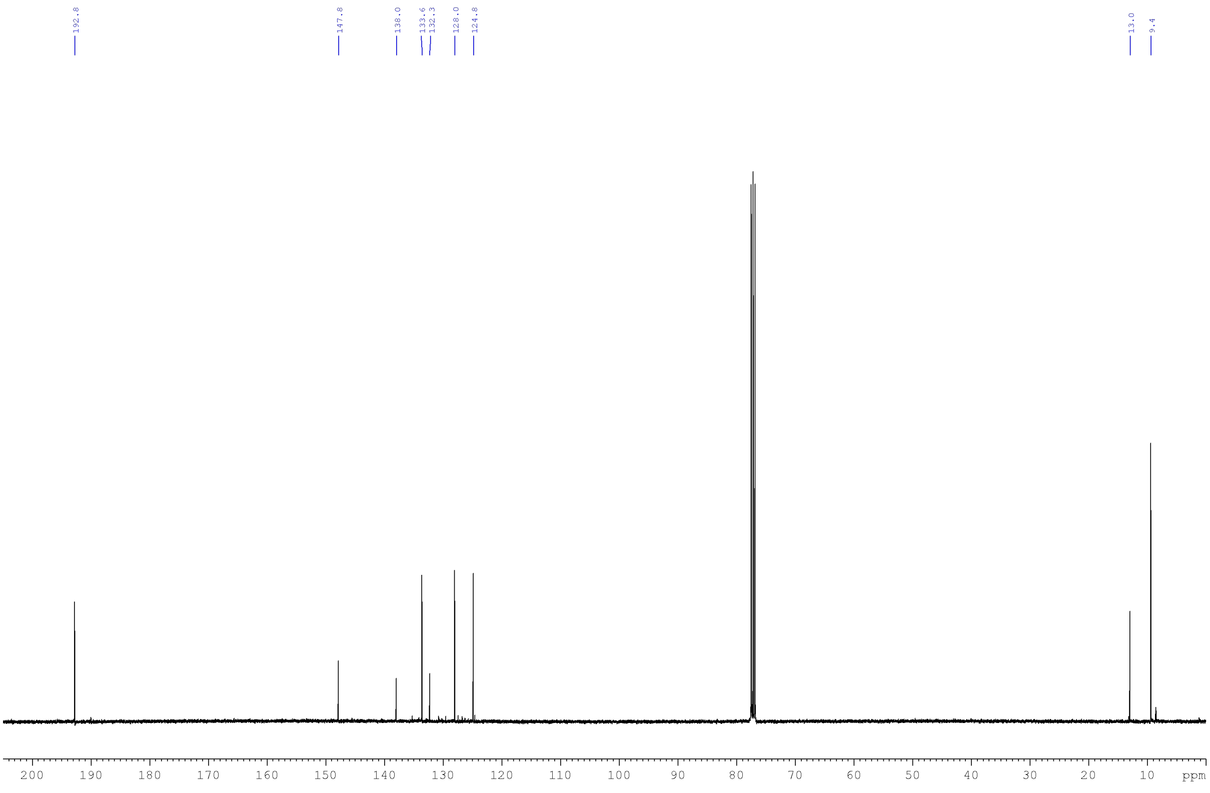
192.8
147.8
138.0
133.6
132.3
128.0
124.8
13.0
9.4
200
190
180
170
160
150
140
130
120
110
100
90
80
70
60
50
40
30
20
10
ppm

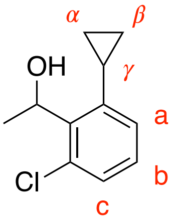
α
β
γ
OH
a
b
Cl
c

11

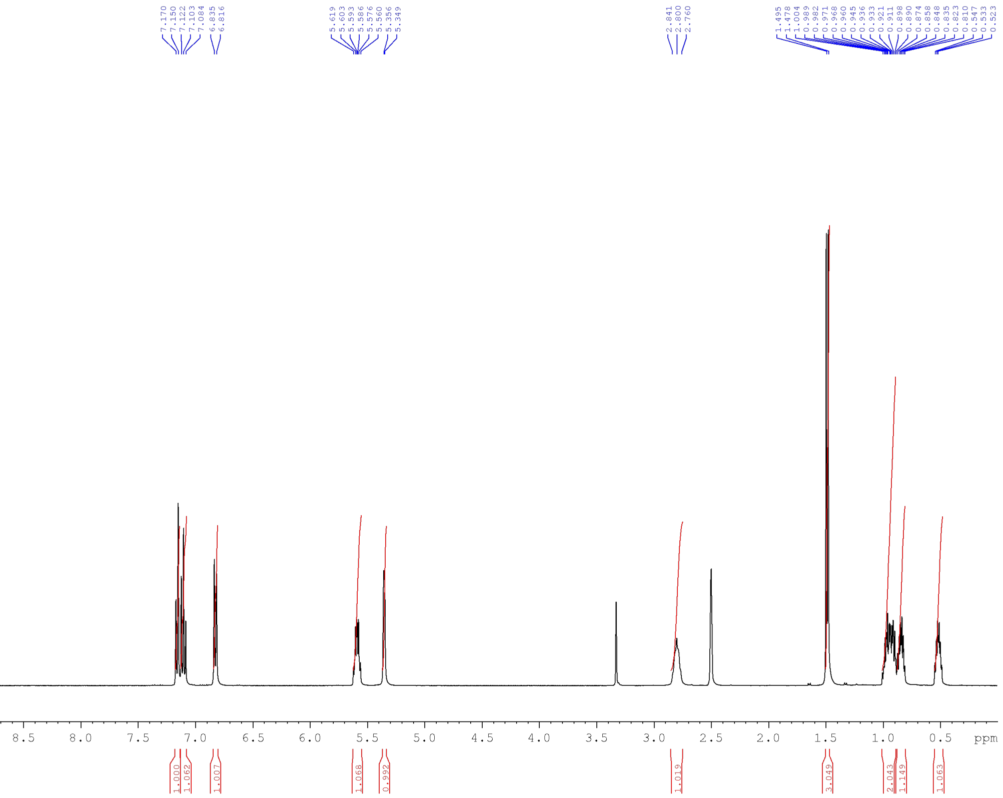
7.170
7.150
7.122
7.103
7.084
6.835
6.816
5.619
5.603
5.593
5.586
5.576
5.560
5.356
5.349
2.841
2.800
2.760
1.495
1.478
1.004
0.989
0.982
0.971
0.968
0.960
0.945
0.936
0.933
0.921
0.911
0.898
0.890
0.874
0.858
0.848
0.835
0.823
0.810
0.547
0.533
0.523
10.0 9.5 9.0 8.5 8.0 7.5 7.0 6.5 6.0 5.5 5.0 4.5 4.0 3.5 3.0 2.5 2.0 1.5 1.0 0.5 ppm
1.000
1.062
1.007
1.068
0.992
1.019
3.049
2.043
1.149
1.063

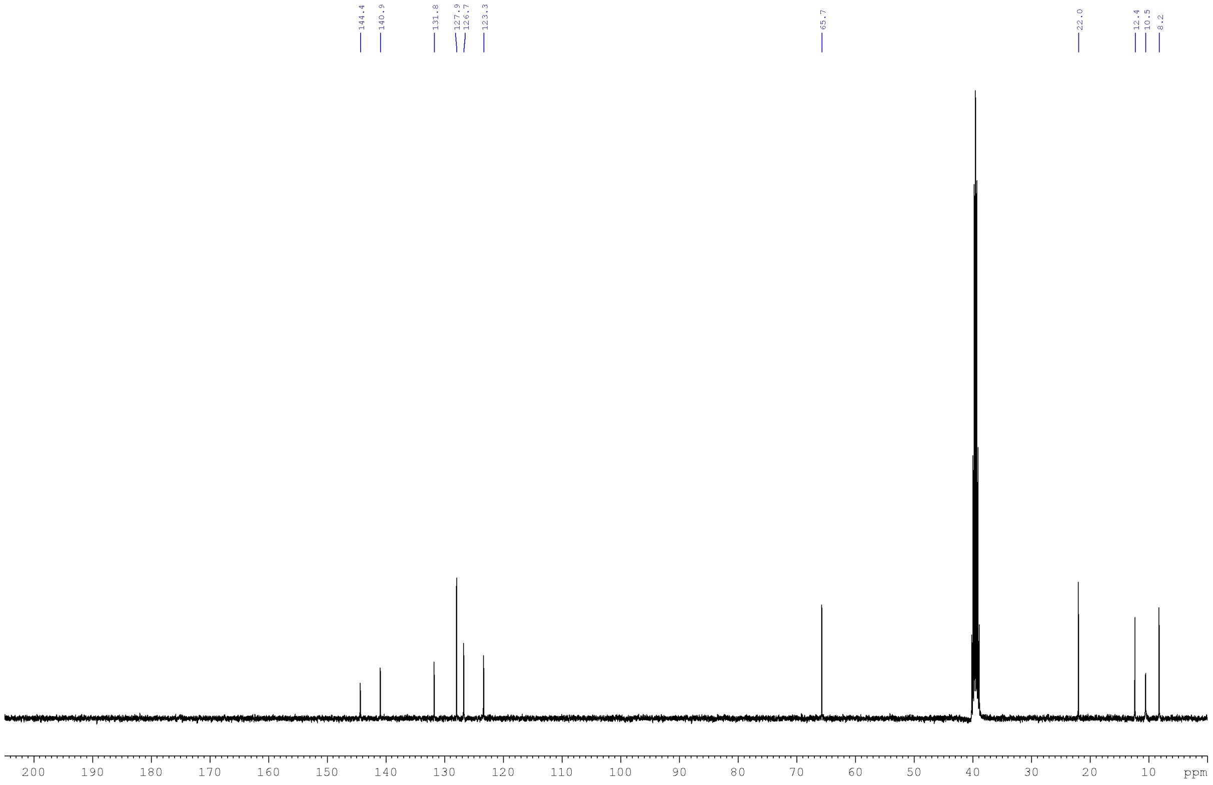
144.4
140.9
131.8
127.9
126.7
123.3
65.7
22.0
12.4
10.5
8.2
200 190 180 170 160 150 140 130 120 110 100 90 80 70 60 50 40 30 20 10 ppm

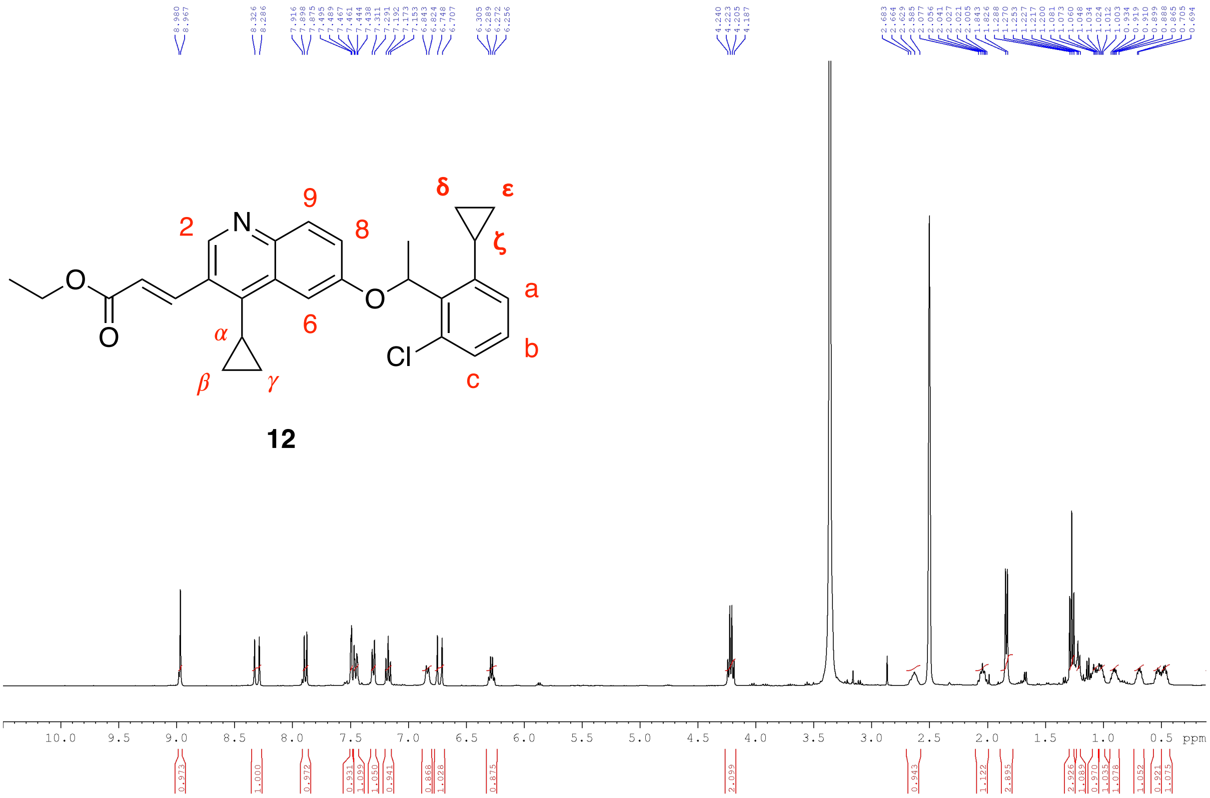
8.980
8.967
8.326
8.286
7.916
7.898
7.875
7.495
7.489
7.467
7.461
7.444
7.438
7.311
7.291
7.192
7.173
7.153
6.843
6.824
6.740
6.707
6.305
6.289
6.272
6.256
4.240
4.223
4.205
4.187
2
N
9
8
O
O
O
α
6
β
γ
δ
ε
ζ
a
b
c
Cl
12
10.0
9.5
9.0
8.5
8.0
7.5
7.0
6.5
6.0
5.5
5.0
4.5
4.0
3.5
3.0
2.5
2.0
1.5
1.0
0.5
ppm

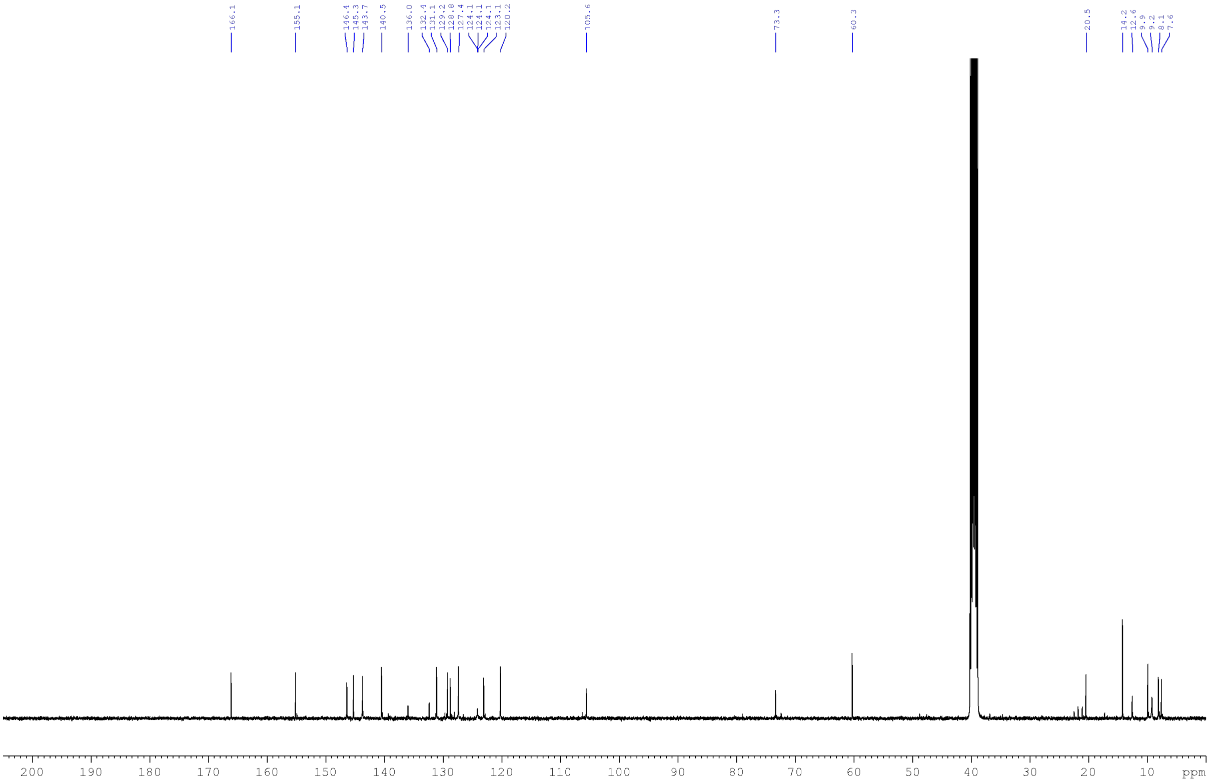
166.1
155.1
146.4
145.3
143.7
140.5
136.0
132.4
131.1
129.2
128.8
127.4
124.1
124.1
123.1
120.2
105.6
73.3
60.3
20.5
14.2
12.6
9.9
9.2
8.1
7.6
200
190
180
170
160
150
140
130
120
110
100
90
80
70
60
50
40
30
20
10
ppm

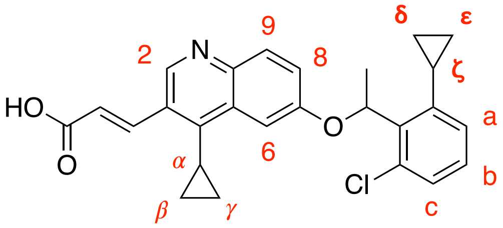

13

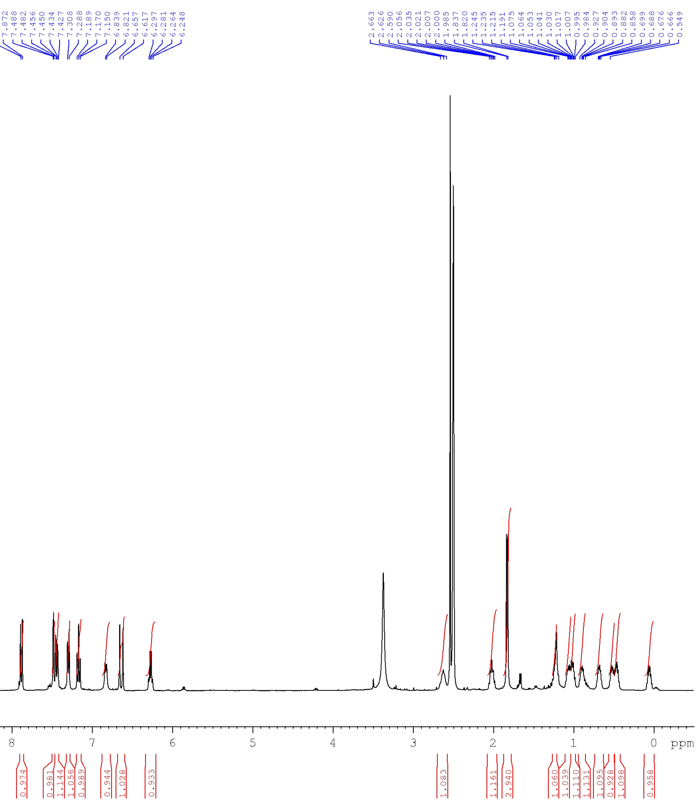

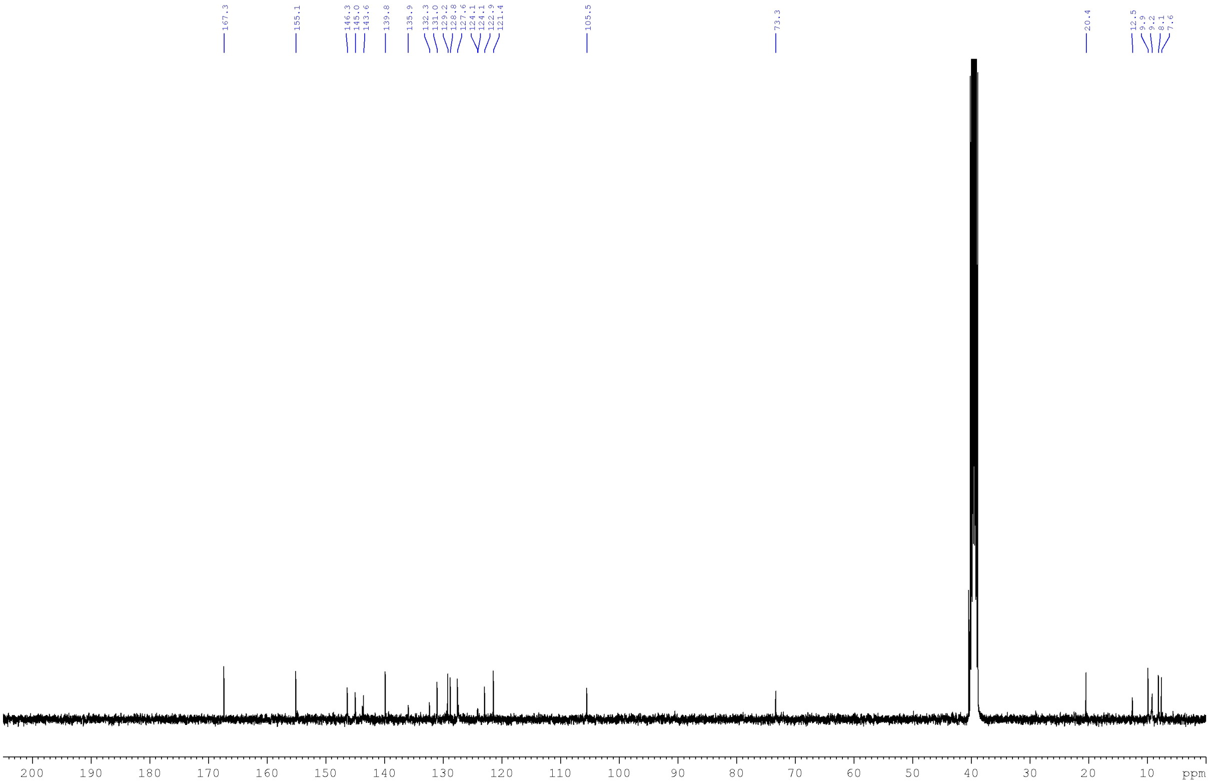

SUPPLEMENTARY REFERENCES

1 Woebking, B. *et al.* Drug-lipid A interactions on the Escherichia coli ABC transporter MsbA. *J. Bacteriol.* **187**, 6363-6369, doi:10.1128/JB.187.18.6363-6369.2005 (2005).

2 Ho, H. *et al.* Structural basis for dual-mode inhibition of the ABC transporter MsbA. *Nature* **557**, 196-201, doi:10.1038/s41586-018-0083-5 (2018).
