# Supplementary material for: Energetics of lipid transport by the ABC transporter MsbA is lipid dependent
Source: Commun Biol. 2021 Dec 9;4:1379. doi: 10.1038/s42003-021-02902-8 (PMC8660845; doi:10.1038/s42003-021-02902-8)
Supplement: Supplementary file 2 — Description of Additional Supplementary Files [file 42003_2021_2902_MOESM2_ESM.pdf]

Description of Additional Supplementary Files

File name: Supplementary Data 1.
Description: Source data for figures and supplementary figures.
